# RSM based optimization and environmental impact assessment of a ternary blended one part alkali activated binder with enhanced mechanical performance

**DOI:** 10.1038/s41598-026-47851-6

**Published:** 2026-04-10

**Authors:** G. S. Gana, Shreelaxmi Prashant, Poornachandra Pandit

**Affiliations:** Manipal Institute of Technology, Manipal Academy of Higher Education, Manipal, India

**Keywords:** Alkali activated materials, RSM, Compressive strength, alkali activation, One part, Activators, Industrial waste, Optimisation, Engineering, Materials science

## Abstract

The performance of AABs is governed by a complex interplay of variables such as binder content, activator dosage, and supplementary materials. Therefore, systematic optimisation is essential to develop mixes that offer both high performance and environmental benefits. The performance of ternary one-part alkali-activated binders incorporating Class F fly ash, GGBS, and silica fume was evaluated and optimised using Box–Behnken–based response surface methodology. BBD was utilised to evaluate and fine-tune the effects of critical parameters such as the binder ratio, solid activator dosage, and silica fume content on compressive strength. Most of the trial mixes achieved strengths exceeding 40 MPa due to the synergistic effect of high GGBS content (30–35%), silica fume (5–10%), and an optimal alkali activator dosage. Among the evaluated mixes, 7.5% silica fume was found to provide the most balanced performance in terms of flowability, consistency, and setting behaviour. The optimised mix achieved compressive strengths of 47 MPa at 7 days and 54 MPa at 28 days, demonstrating its potential for structural elements, repair or retrofitting applications where compressive strength above 50 MPa is required. BBD was employed to model and optimise compressive strength. The developed quadratic model was statistically significant (*p* = 0.037; R² = 90.91%) with insignificant lack-of-fit (*p* > 0.05). The experimental validation showed good agreement between predicted and measured values, with errors below 10%, confirming the reliability of interpolation within the investigated domain.

## Introduction

The global construction industry greatly contributes to environmental pollution, mainly due to the extensive use of Ordinary Portland Cement (OPC). The production of OPC is thought to be responsible for 8% of the world’s CO_2_ emissions. The increasing sustainability concerns and the growing demand for infrastructure have clearly driven demand for environmentally responsible construction materials that reduce impact on the environment without compromising performance. Recently, research has increasingly focused on the use of alternative constituents, including supplementary binders, aggregates, and water sources, along with chemical admixtures and improved processing techniques, to enhance the efficiency and durability of concrete^1,2^. Alkali-activated materials (AAMs) are gaining importance in research as sustainable binders, offering a better alternative to traditional Portland cement by employing various precursors^3^. In recent years, AAMs have been widely investigated using a variety of precursors and alkaline activators^4^. Recent developments in low-energy synthesis techniques and increased utilization of industrial by-products are enhancing the feasibility of these systems for large-scale construction applications^5–8^. The majority of prior investigations have primarily focused on conventional two-part alkali activation systems, which involve the separate addition of liquid alkaline solutions.

The two-part alkali-activated system process involves preparation and mixing of two components: a solid aluminosilicate precursor and a liquid alkaline activator solution. In this system, the activator is pre-dissolved in water and then combined with the precursor at the time of mixing to initiate geo-polymerisation or alkali activation^9,10^. AAMs are produced by activating aluminosilicate or calcium-aluminosilicate precursors under strongly alkaline conditions^11^. Many types of industrial and agricultural wastes are utilised as precursors, such as fly ash, GGBS, Bagasse ash, and clay^12,13^. The two-part system offers superior mechanical and reaction performance but is less user-friendly due to handling liquid alkali^14^. In contrast, the one-part alkali activation method, which involves dry-blending solid activators with precursors and adding water during blending, has gained attention in recent years, offering easier handling and safer application, making it attractive for practical and on-site use. Both systems produce similar reaction products and microstructures, but the two-part system reaches a higher reaction extent and strength^10,15^.

The one-part AAM involves dry blending of Precursors and solid alkali activators, followed by the addition of water, similar to cement mixing. This approach effectively overcomes the disadvantages of the two-part AAB, enabling ease in cast-in-situ construction applications. In OPAAB, common precursors include GGBFS, fly ash, metakaolin, nickel slag, lithium slag, and various types of biomass or agricultural ashes^16,17^. The choice of precursor significantly affects strength, setting time, and durability^18–22^. Similarly, Sodium metasilicate, Sodium hydroxide, sodium carbonate, potassium hydroxide, and waste-derived powders (e.g., rice husk ash, calcined oyster shell) are typical activators that are used in OPAAB. The type and alkali activator percentage have its influence on reaction kinetics, microstructure, and mechanical properties^23–26^. Therefore, careful selection of both precursors and activators, along with their relative proportions, is essential to optimise performance and ensure materials suitability for practical applications. Optimising materials is very important for achieving desired results, but it often relies on trial and error, lacking precision.

Alkali-activated binders exhibit complex behaviour influenced by multiple variables, including precursor composition, activator nature and concentration, curing conditions, and water-to-binder ratio. These factors interact nonlinearly, making it difficult to predict performance without a structured approach. Optimisation tools play a crucial role in developing the mix design of alkali-activated binders, enabling the systematic identification of optimal mix proportions and processing conditions to achieve desired material properties, such as compressive strength, workability, and durability, while minimising costs and environmental impact.

Response Surface Methodology (RSM), a statistical optimisation tool, can be used to address this complexity by modelling the relationships between input variables and responses(binder proportion, activator percentage, etc.), allowing for the identification of an optimal mix with fewer experiments compared to traditional trial-and-error methods. By using RSM, researchers can efficiently explore the design space, quantify the effects of variables and their interactions, and achieve a balance among performance, cost, and sustainability. RSM provides a mathematical model to predict performance based on input variables and identifies significant interactions between variables that may not be apparent in single-factor studies^27–29^.

Recent applications of RSM in alkali-activated systems have primarily focused on optimising specific performance parameters. Studies have demonstrated the use of RSM to enhance mechanical performance in one-part systems incorporating advanced additives such as recycled polymers and graphene^30^, as well as to optimise activator composition in MgO-based composite systems^31^. Box–Behnken design has been employed to evaluate multi-objective responses in waste-derived alkali-activated mortars^32^, primarily to improve modelling efficiency within restricted experimental domains. Similarly, statistical optimisation has been applied to slag-dominant concrete systems incorporating recycled aggregates and silica fume^33^. Beyond binary and slag-based systems, Central Composite Design has been used to model compressive strength in rice husk ash-based binders^34^, while broader sustainability-driven optimisation was studied^35^. The objective is to utilise the RSM optimisation tool and ascertain the ideal ratio of mixing for the considered precursors and activators using the Box-Behnken design (BBD) method. This allows for a comprehensive assessment of the individual and interactive effects of these variables on the overall performance of OPAAB mixtures. This study addresses this gap by applying the Box-Behnken Design (BBD) within Response Surface Methodology (RSM) to optimise an OPAAB formulated with a ternary binder and solid activators, aiming to achieve higher strength, thereby advancing low-carbon construction.

### Research significance

Although one-part alkali-activated binders have attracted increasing attention for their practical and safety advantages over conventional two-part systems, existing studies predominantly focus on binary binder combinations or rely on empirical, trial-and-error mix-design approaches. Recent studies on RSM optimisation focus either on binary precursor combinations or on activator optimisation alone. Systematic statistical modelling of ternary binders(FA+GGBS + SF) incorporating dual alkali activators remains limited. Furthermore, limited attention has been given to the simultaneous evaluation of fresh and hardened properties within a unified optimisation framework. The present study employs BBD to investigate interaction behaviour and optimisation of a ternary alkali-activated binder system.

## Materials

### Precursors

**Fig. 1:**
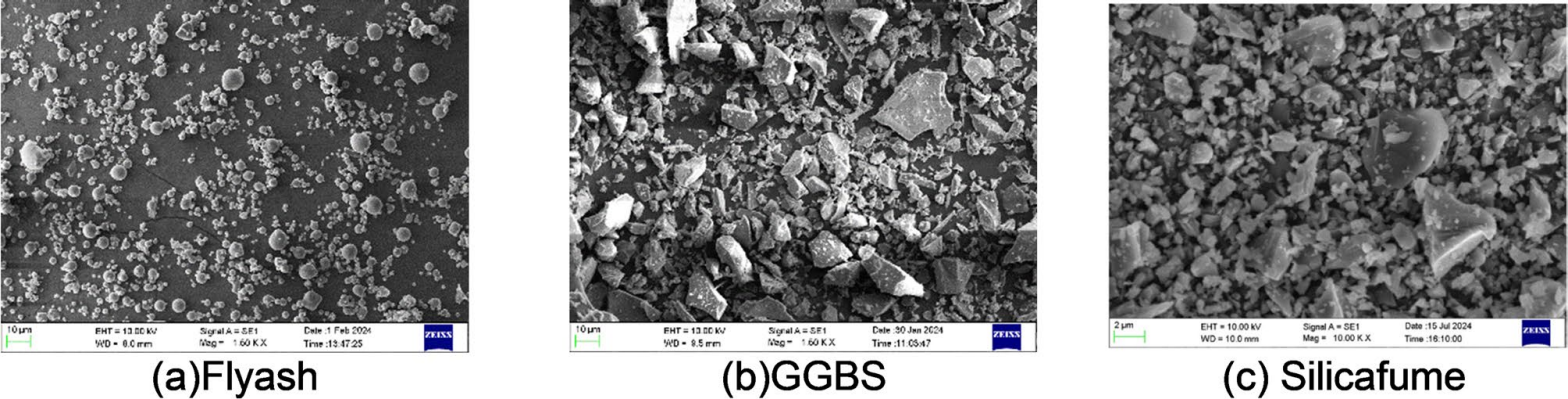
SEM images of binders. (**a**) Flyash, (**b**) GGBS, (**c**) Silicafume.

**Fig. 2:**
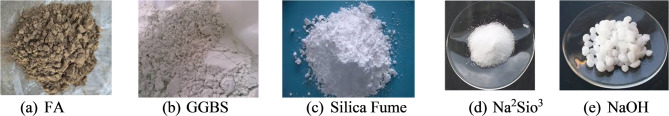
Images of Binder and alkali activators.

Class F fly ash, GGBS and Silica fume are used as precursors in the experiment. FA conforming to ASTM C618 and IS 3812 (Part 1)^36,37^ was utilised, procured from the Thermal Power Plant, Bellary and Ground Granulated Blast Furnace Slag (GGBS) was sourced from JSW Steels, Mangalore, and complies with ASTM C989^38^. Silica fume(white) was procured from Astra Chemicals, Chennai, as shown in Fig. 2. The oxide composition of the binders is presented in Table 1. The microstructural characteristics of the precursor materials were examined using scanning electron microscopy (SEM), ZEISS EVO MA18, as illustrated in Fig. 1. As shown in Fig. 1(a), fly ash exhibits predominantly spherical morphology with fine and smooth surface, while GGBS (Fig. 1(b)) exhibit irregular morphology with sharp edges and rough surface.

**Table 1:** Chemical composition of Fly ash and GGBS.

Component composition (%)	FA	GGBS	Silica fume
SiO_2_	55.8	37.30	95.8
Al2O_3_	28.2	16.60	1.98
Fe_2_O_3_	7.45	0.37	0.71
TiO_2_	2.92	0.82	–
CaO	1.54	34.70	0.61
MgO	0.44	6.87	–
Na_2_O	1.23	0.63	–
Loss of Ignition	1.75	–	1.19

### Solid activators

Sodium Hydroxide (NaOH): Anhydrous sodium hydroxide pellets were utilised as one of the primary alkaline activators. It consists of 99.76% and 0.21% by mass of sodium hydroxide and sodium Carbonate, respectively (provided by the manufacturer).

Sodium Metasilicate (Na₂SiO₃): In order to improve the geopolymerization process, anhydrous sodium metasilicate powder was also employed as a co-activator. It has a total alkalinity of 29% by mass of Na_2_O. Both activators were procured in anhydrous form from Astraa Chemicals, Chennai (Fig. 2).

### Mix proportion and samples preparation

**Fig. 3:**
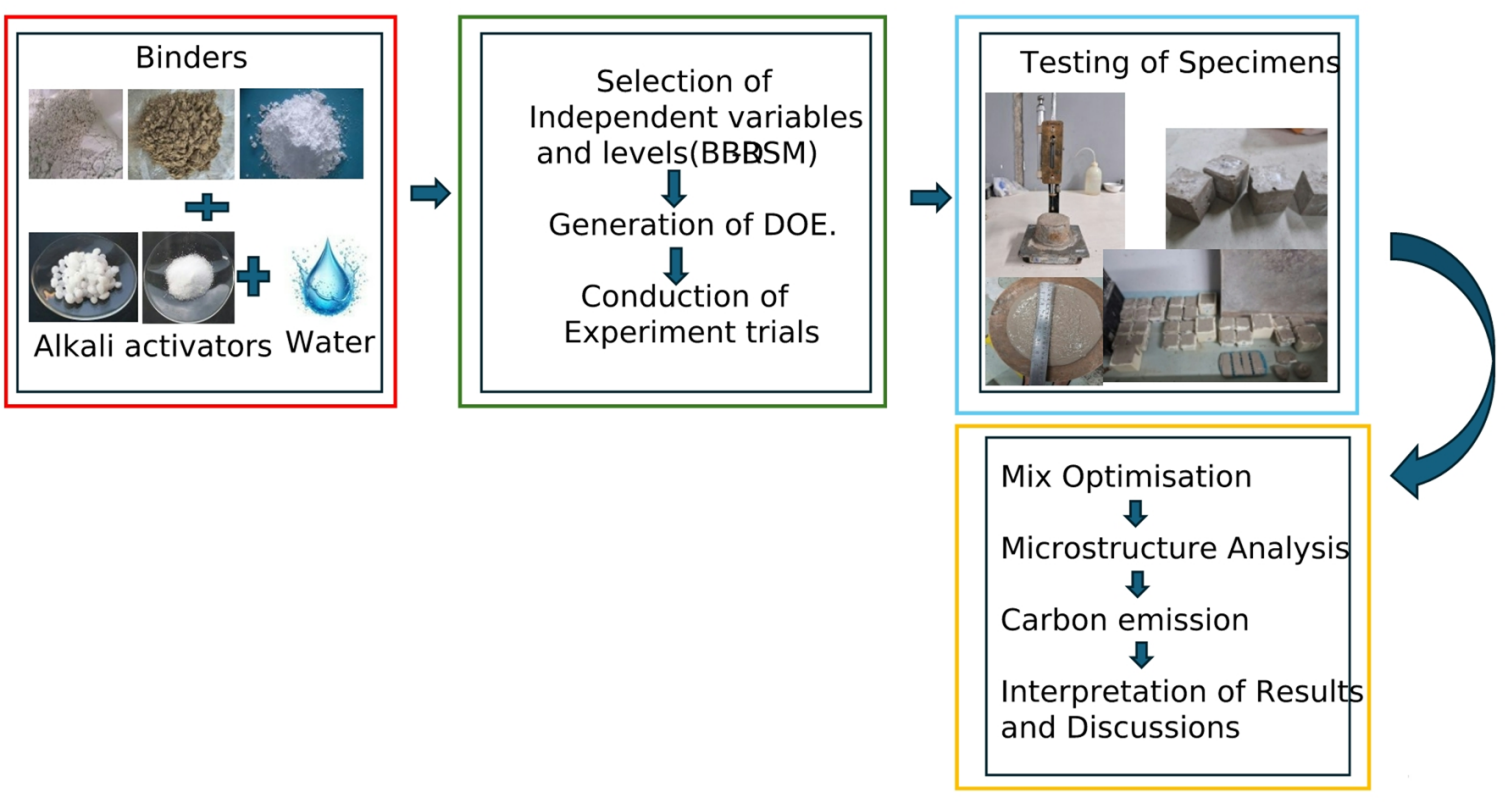
Flow diagram of OPAAB methodology.

The One-Part Alkali-Activated Binder (OPAAB) was prepared using fly ash, GGBS, and Silica fume as precursors, activated with anhydrous sodium metasilicate and sodium hydroxide. The ratio of NaOH-to-Na₂SiO₃ was maintained at 1:2 in the experiment. Initially, the required quantities of precursors are weighed and dry-blended in a mortar mixer. Then, the calculated quantity of activator in dry form is added to the mixer and mixed until the mix appears homogeneous and uniform. The predetermined water is then added to the mix, followed by continued mixing at 120 rpm for 3–4 min until a consistent and homogeneous paste is achieved. The fresh paste was assessed for flow characteristics, ASTM C1437, Consistency in accordance with IS 4031 (Part 4):1988^39^, and setting time with initial and final settings recorded at 5-minute and 30-minute intervals, respectively, following ASTM C191–21^40^. Subsequently, the mixes were cast into 50 mm cube PVC moulds. For each mix, 6 cubes were cast and cured under ambient air conditions until testing. Compressive strength tests were conducted at 7 and 28 days in accordance with ASTM C109^41^. The flow diagram of the OPAAB methodology is shown in the Fig. 3.

The experimental matrix was developed by systematically varying key parameters governing the reaction kinetics and the performance of one-part alkali-activated binders. The fly ash content was varied from 50% to 75% by weight of the total solid binder. This range was selected to ensure sufficient calcium contribution from GGBS for ambient curing while maintaining fly ash as the dominant precursor to control reaction rate and workability. Fly ash contents below 50% tend to result in GGBS-dominated systems with rapid setting, whereas fly ash contents exceeding 80% often exhibit reduced early reactivity and strength development under ambient curing conditions^21,42,43^.

The solid alkali activator dosage was varied at 10%, 12%, and 14% by weight of the total solid binder. These levels were selected based on previous studies and trial-and-error testing. Thus, indicating that activator dosages below 10% are insufficient to achieve effective dissolution of aluminosilicate precursors, while dosages above 14% may lead to excessive alkalinity, rapid gel formation, and deterioration of workability in one-part systems^44^. A constant water-to-binder ratio (w/b) of 0.30 was maintained for all mixtures. In one-part alkali-activated binder systems, water primarily serves as a reaction medium, facilitating the dissolution of aluminosilicate species and their subsequent polycondensation. Preliminary trials indicated that a w/b ratio of 0.30 provided adequate workability while maintaining sufficient alkalinity concentration for effective geopolymerization. Therefore, to avoid confounding effects during Response Surface Methodology (RSM) optimization of precursor proportions and activator dosage, the w/b ratio was kept constant throughout the study. Detailed mix proportions are provided in Table 3, with mixes designated as M1, M2, etc., for clarity.

## Optimisation method

### Response surface methodology (RSM)

RSM enables the systematic evaluation of the influences and interfaces of multiple input factors, facilitating the identification of optimal conditions for a desired outcome. This is achieved through the design and analysis of experiments, fitting empirical models (typically second-order polynomials), and using these models to predict and optimize the response variable within a defined experimental^45,46^.

**Fig. 4:**
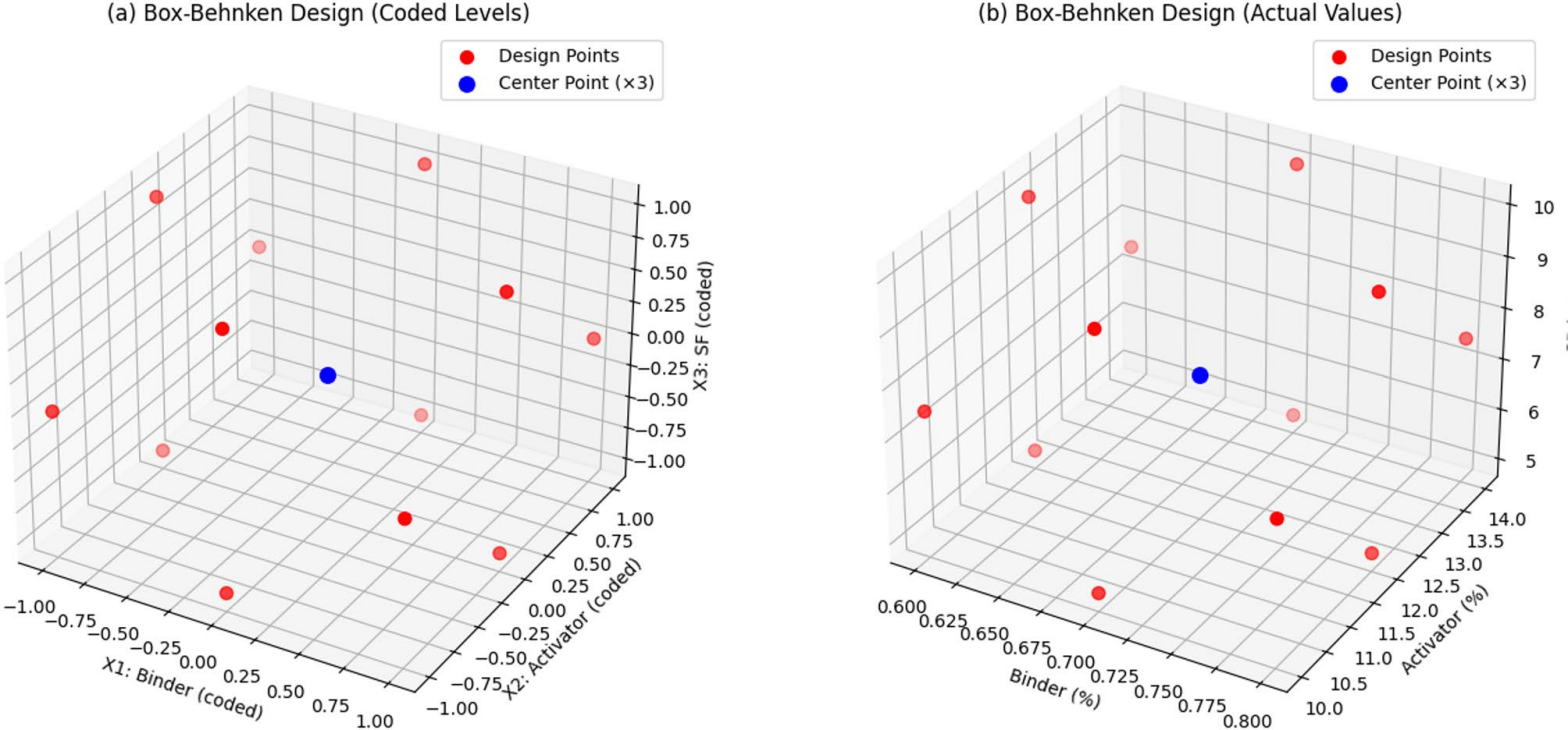
Box-Behnken design for OPAAB: (**a**) coded levels, and (**b**) actual values of OPAAB (%).

In this study, RSM was employed to evaluate the combined influences of independent variables (binder, activator) and to establish functional relationships with the output responses (Compressive strength, flow, setting time). For this purpose, different statistical models were employed within the response surface framework to identify the relationships between the measured responses and the independent variables. Among the available designs, the Box–Behnken Design (BBD) is a widely adopted approach in RSM. The geometric structure of the BBD in three-dimensional space, along with the arrangement of coded design points, is illustrated in Fig. 4. In this, the central, high, and low levels of each factor are denoted as 0, + 1, and − 1, respectively. Experimental runs were conducted at each of these design points to generate the dataset required for surface analysis. Using these data, RSM applies nonlinear regression to derive the predictive model. Typically, the major and interacting effects of the factors are described by a second-order polynomial, which makes it possible to identify the ideal conditions.

BBD is specifically tailored for constructing quadratic models without requiring experiments at the extreme corners of the factor space. BBD was preferred over CCD for optimisation as it avoids extreme factor combinations that may produce unstable mixes in one-part alkali-activated systems, while efficiently capturing interaction and quadratic effects with fewer experiments. BBD tests each factor at three levels and efficiently estimates both main and interaction effects, while reducing the number of test runs compared to complete factorial designs. This design is particularly advantageous for process optimization as it avoids potentially unsafe or impractical combinations of factor extremes and provides reliable predictions of optimal conditions^47^.

### Mix proportions derived from RSM

- Coded Space Cube (–1, 0, + 1): Shows the experimental points of Box-Behnken Design in normalized (coded) form. This is useful for statistical analysis and RSM fitting in software like Minitab.
- Actual Factor Space: Shows the same points translated to real-world values: Binder: GGBS replacement-20–40%, Activator: 10–14% ,Silica Fume: 5–10%,w/b ratio = 0.3 (Tables 2 and 3).

**Table 2:** Box Behnken method for OPAAB.

Variable factors	Code	Level		
		− 1	0	1
Binder(GGBS replacement) (%)	X1	20	30	40
Alkali Activator (%)	X2	10	12	14
Silica fume (%)	X3	5	7.5	10

**Table 3:** Mix design derived for OPAAB by BBD.

Mix No.	FA (%)	GGBS (%)	Silica fume (%)	Alkali activator (%)
1	52.5	40	7.5	10
2	72.5	20	7.5	10
3	52.5	40	7.5	14
4	72.5	20	7.5	14
5	55	40	5	12
6	75	20	5	12
7	50	40	10	12
8	70	20	10	12
9	65	30	5	10
10	65	30	5	14
11	60	30	10	10
12	60	30	10	14
13	62.5	30	7.5	12
14	62.5	30	7.5	12
15	62.5	30	7.5	12

## Results and discussions

### Fresh properties

#### Consistency

**Fig. 5:**
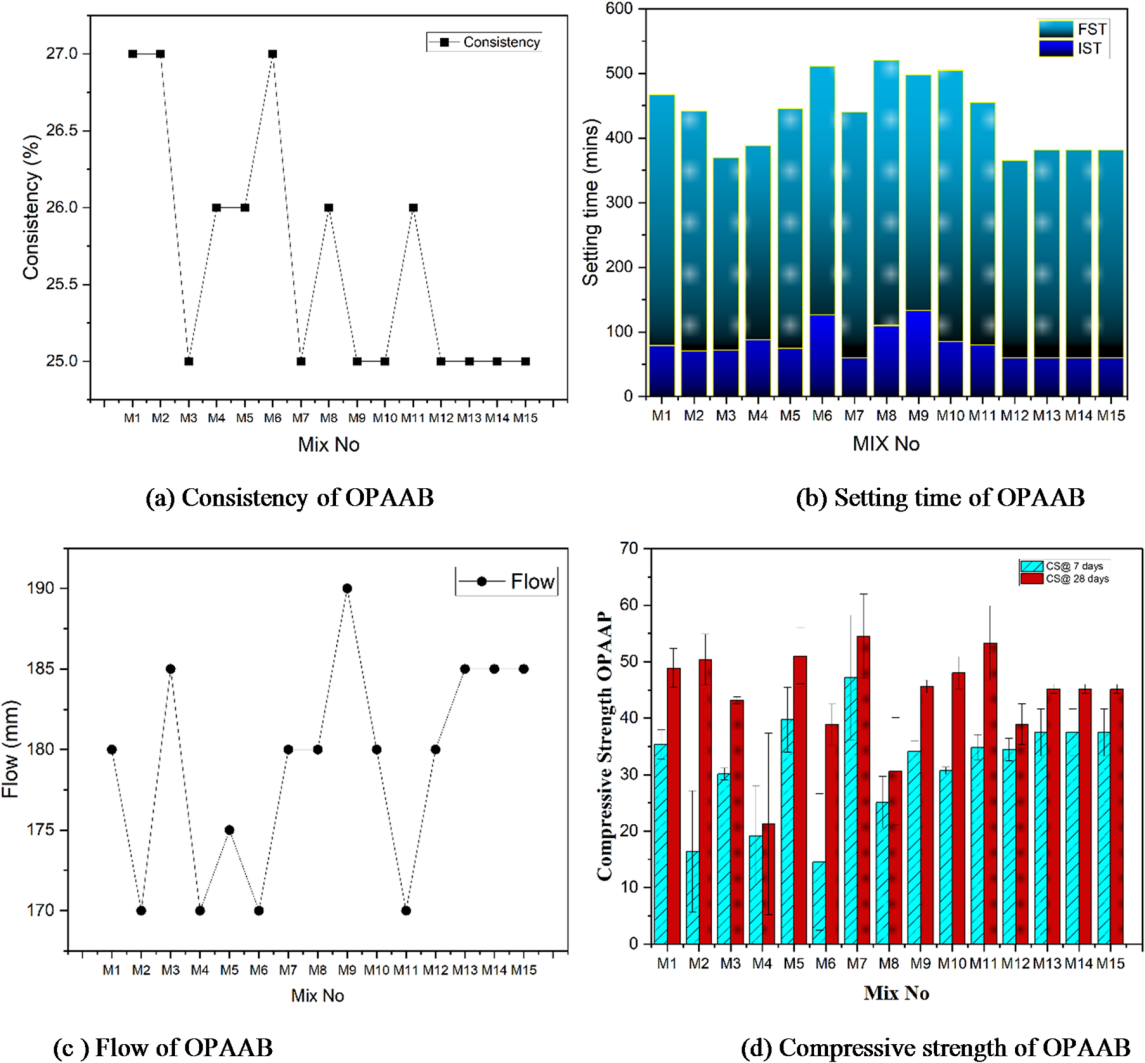
Fresh and Hardened property results of OPAAB.

The consistency of the one-part alkali-activated binder, as represented in Fig. 5(a), ranged narrowly between 25% and 27% across all mixes, indicating a high degree of uniformity in fresh-state behaviour. Variations in the activator dosage and GGBS level are responsible for minor consistency differences among the mixes. Higher activator dosages (M3, M4, M10, M12) increase ionic concentration and early reaction kinetics, while increased GGBS content enhances calcium availability, both of which can marginally influence paste stiffening at the fresh stage. Similar observations report that a constant water-to-binder ratio and gradual activator dissolution resulted in stable and reproducible fresh properties^48,49^. However, the narrow range may reduce the sensitivity of the model for this parameter, but it also indicates good experimental control and mix stability. The interaction was maintained in the model to examine subtle interaction effects among precursors.

#### Setting time

As displayed in Fig. 5(b), the initial setting time ranged from 60 to 133 min, with Mixes 7, 12, 13, 14, and 15 exhibiting the shortest initial setting time of 60 min and Mix 9 the longest at 133 min. These mixes exhibit faster early geopolymerization, likely due to the high reactivity of GGBS, silica fume, combined with optimised activator levels. The final setting time varied between 298 and 420 min, with Mix 3 showing the earliest final set at 298 min and Mix 10 the latest at 420 min. The variation in setting times is linked to differences in binder reactivity and alkaline activator content. Final setting times mark the completion of hardening, with Mix 3 showing the fastest set, while longer times in Mixes 8, 9, 10, and 1 are associated with higher fly ash and lower silica fume content, which delay geopolymerization. The narrow consistency range facilitates consistent dissolution of solid activators and controlled early geopolymerisation kinetics. Such stability in the fresh state is known to reduce variability in early-age reactions and promote predictable setting behaviour in OPAAB^21,49^.

#### Flow

Figure 5(c) shows the flow behaviour of the OPAAB mixes measured in accordance with IS 4031 (Part 4): 1988. The flow values ranged from 170 to 190 mm, indicating consistent workability across all mixes. Mixes 2, 4, and 6 (high fly ash, low GGBS) showed lower flow due to limited calcium availability and reduced activator–binder compatibility, resulting in incomplete early dissolution of aluminosilicate phases and increased internal friction. Mix 9 exhibited the highest flow, likely due to improved particle dispersion associated with an optimised GGBS content, lower silica fume, and lower activator dosage. Mixes 1, 7, 8, 10, and 12 recorded flow values around 180 mm, while the remaining mixes showed intermediate flow due to the combined effects of silica fume filling, GGBS-induced cohesiveness, and controlled activator dissolution^50,51^.

### Mechanical properties

#### Compressive strength

The compressive strength of the 15 RSM-derived OPAAB mixes was evaluated at 7 and 28 days using 50 mm cube specimens (Fig. 5(d)). The 7-day strengths ranged from 14.5 MPa (Mix 6) to 47.2 MPa (Mix 7), while the 28-day strengths varied between 21.2 MPa (Mix 4) and 54.5 MPa (Mix 7). Mix 7 consistently exhibited the highest strength, which can be attributed to an optimal balance of reactive precursors and activator dosage. The combined presence of silica fume and GGBS contributed to strength development by refining pore structure and promoting the formation of geopolymeric N-A-S-H and calcium aluminosilicate hydrate (C-A-S-H) gels, as also evidenced by SEM observations (Fig. 10(g)) and reported in the literature^52^. Mix 4 recorded the lowest 28-day strength (21.2 MPa), potentially due to suboptimal activator content or lower reactivity of the fly ash. In the SEM image (Fig. 11(d)), we can see the dissolution of precursor particles and premature gel precipitation. The precursors require sufficient alkalinity to initiate geopolymerization, and an activator dosage that results in the dissolution of SiO₂ and Al₂O₃, reducing gel formation, as mentioned by the author Duxson in his work^53^. Additionally, excessive silica fume may have increased the Si/Al ratio, leading to a less cross-linked gel structure, which negatively impacts strength as seen by author Fernandes^54^. The rise in compressive strength between 7 and 28 days reflects continued dissolution of aluminosilicate precursors and progressive polymerization of N-A-S-H and C-(A)-S-H gels, a well-established behaviour in fly ash– and GGBS-based alkali-activated binder^53,55^. Mix 6’s (Fig. 11(f)) significant gain suggests delayed reactivity, possibly due to slower dissolution of fly ash particles, which continue to form geopolymeric gels over time^56^. Mixes 13–15 are replicate mixes that showed strengths (37.4 MPa at 7 days, 45.1 MPa at 28 days), reflecting consistent precursor and activator ratios, ensuring uniform reaction kinetics. Variations in binder percentages and surface area of precursors influence reactivity and strength development, as Provis mentions in his work^57^ The water-to-binder ratio, indirectly reflected in consistency (25–27%), also affects porosity and strength, with lower ratios typically enhancing mechanical properties, as mentioned by the authors Hardjito^58^. These results emphasise the possibilities of one-part alkali-activated binder for sustainable construction, with Mix 7 offering high compressive strength due to optimised geopolymerization. Such optimisation of precursor chemistry, activator dosage, and water availability is well recognised to enhance strength development in alkali-activated binders^53,59^. Silica fume also reduces environmental impact by enabling lower alkali-activator dosages to achieve the same mechanical performance.

### Response development from BBD using RSM

#### The residual analysis across parameters

**Fig. 6:**
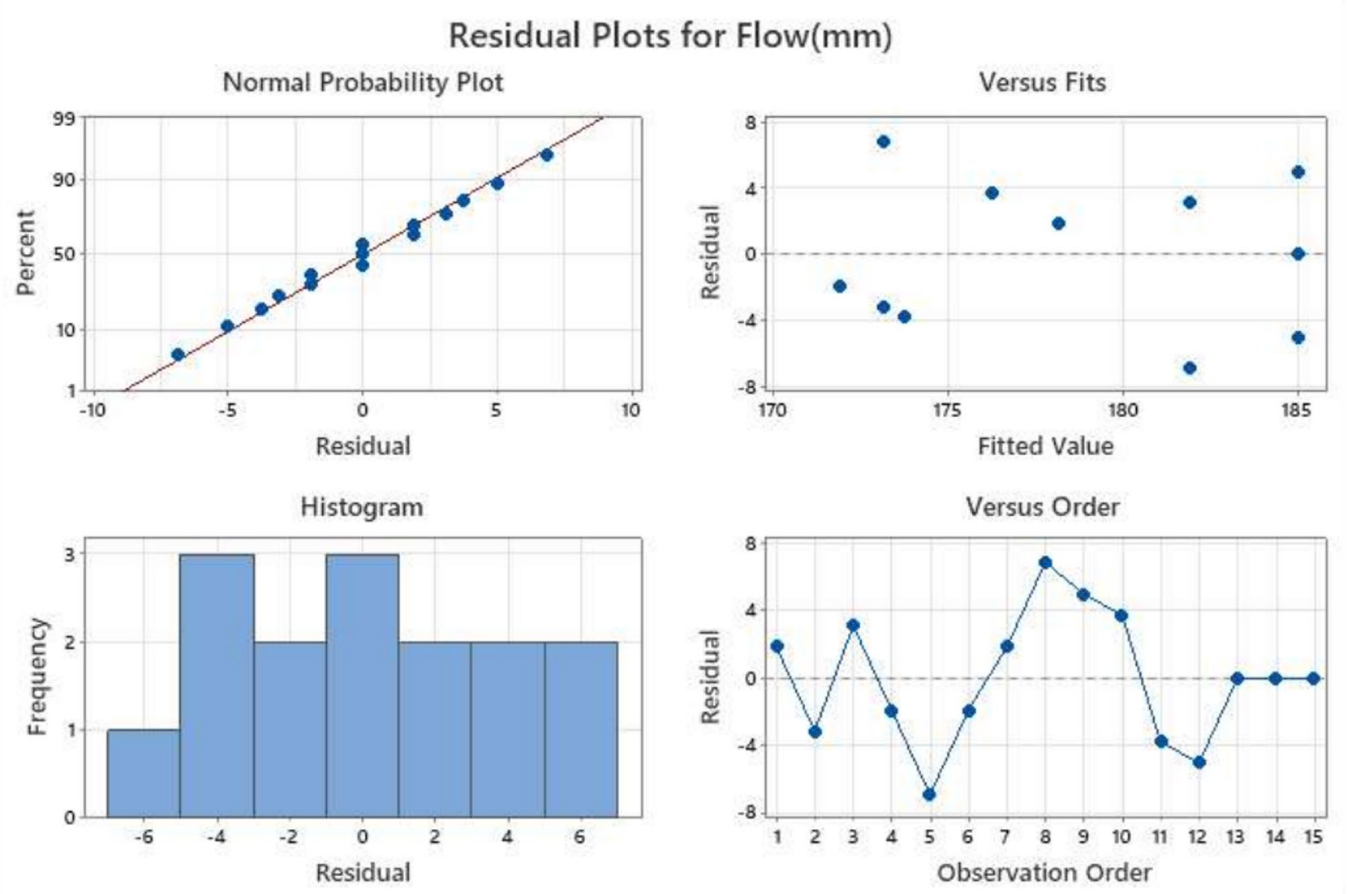
Residual diagnostic plots for flow response showing (**a**) normal probability plot, (**b**) residuals versus fitted values, (**c**) histogram of residuals, and (**d**) residuals versus observation order.

**Fig. 7:**
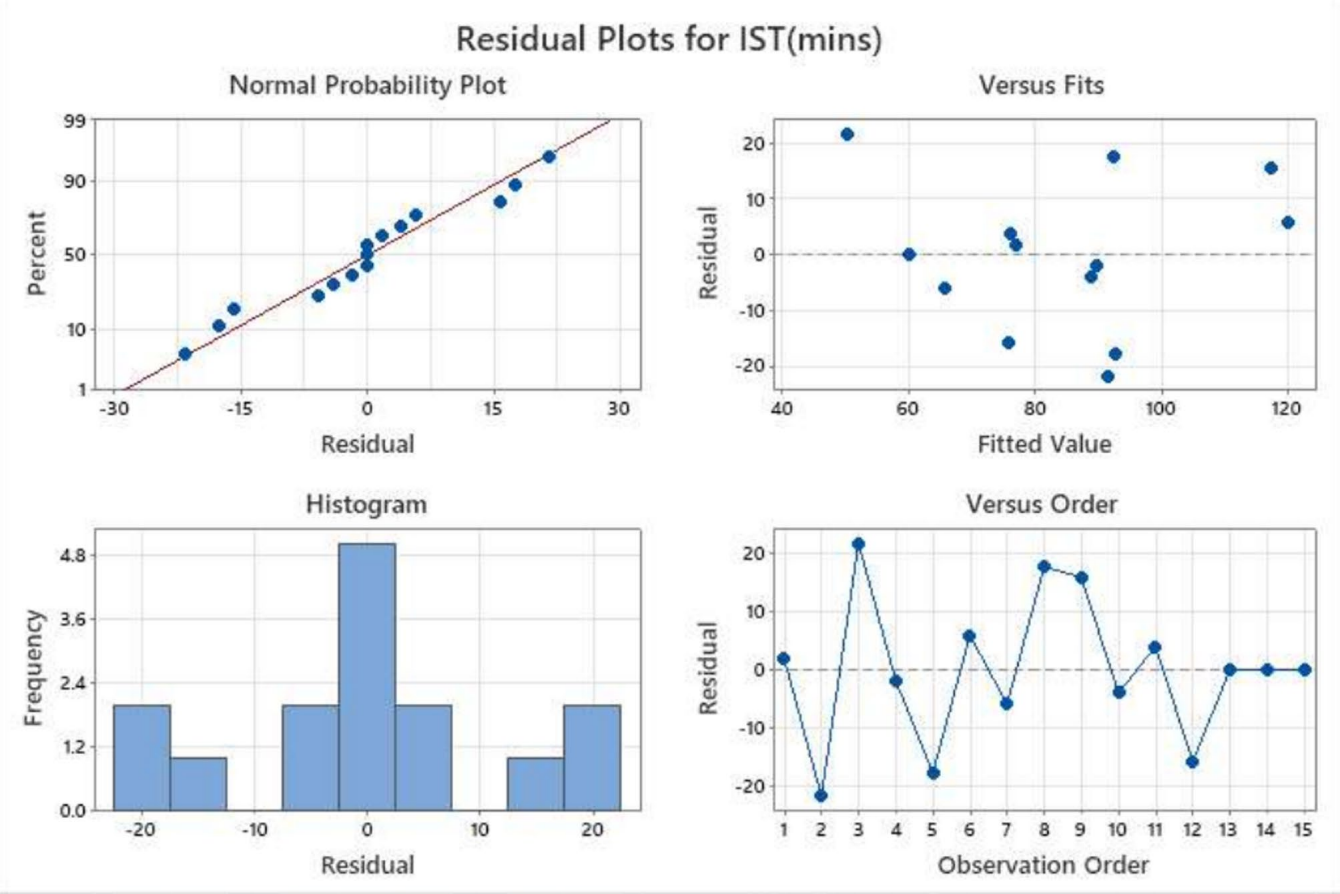
Residual diagnostic plots for initial setting time (mins) response showing (**a**) normal probability plot, (**b**) residuals versus fitted values, (**c**) histogram of residuals, and (**d**) residuals versus observation order.

**Fig. 8:**
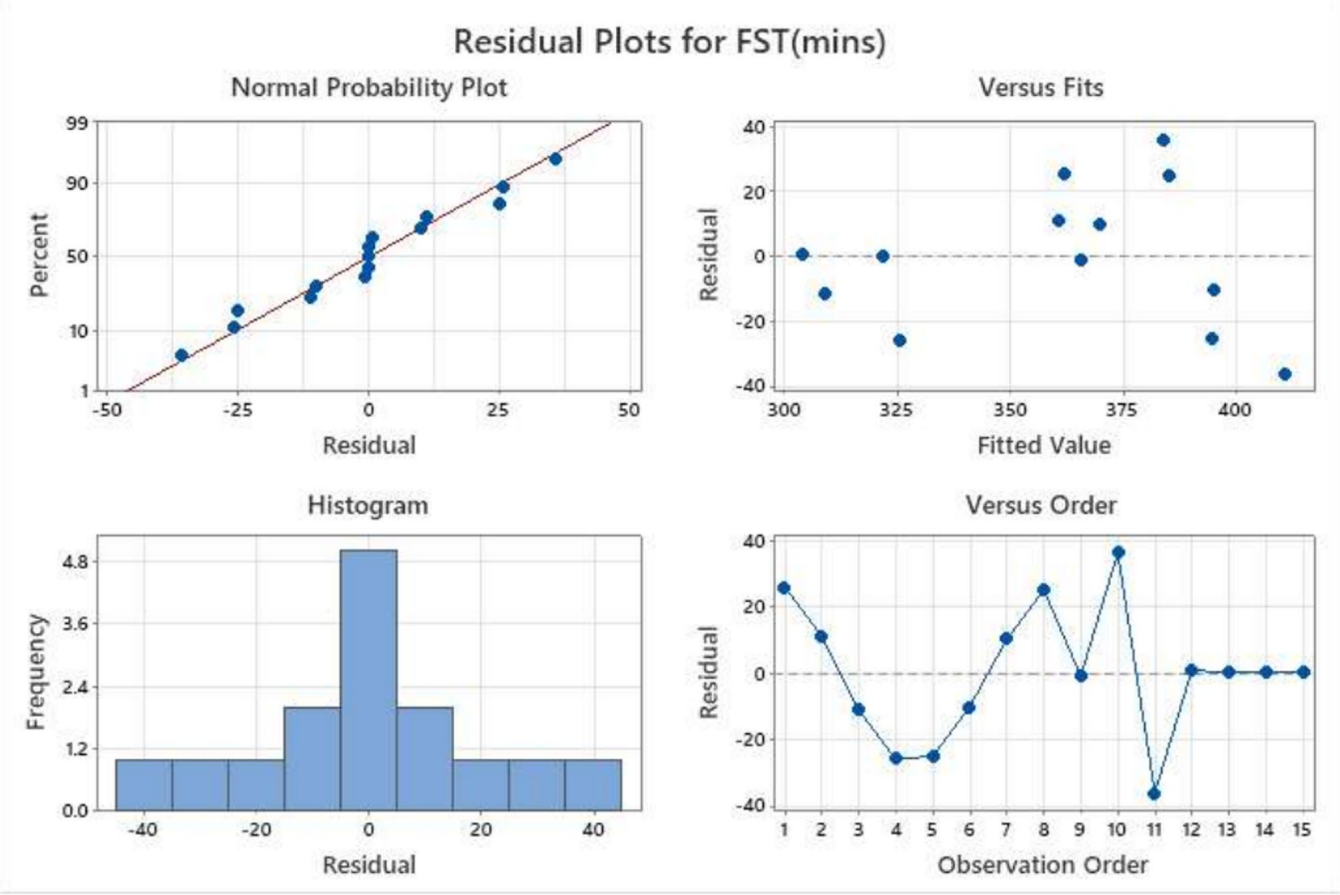
Residual diagnostic plots for final setting time (mins) response showing (**a**) normal probability plot, (**b**) residuals versus fitted values, (**c**) histogram of residuals, and (**d**) residuals versus observation order.

**Fig. 9:**
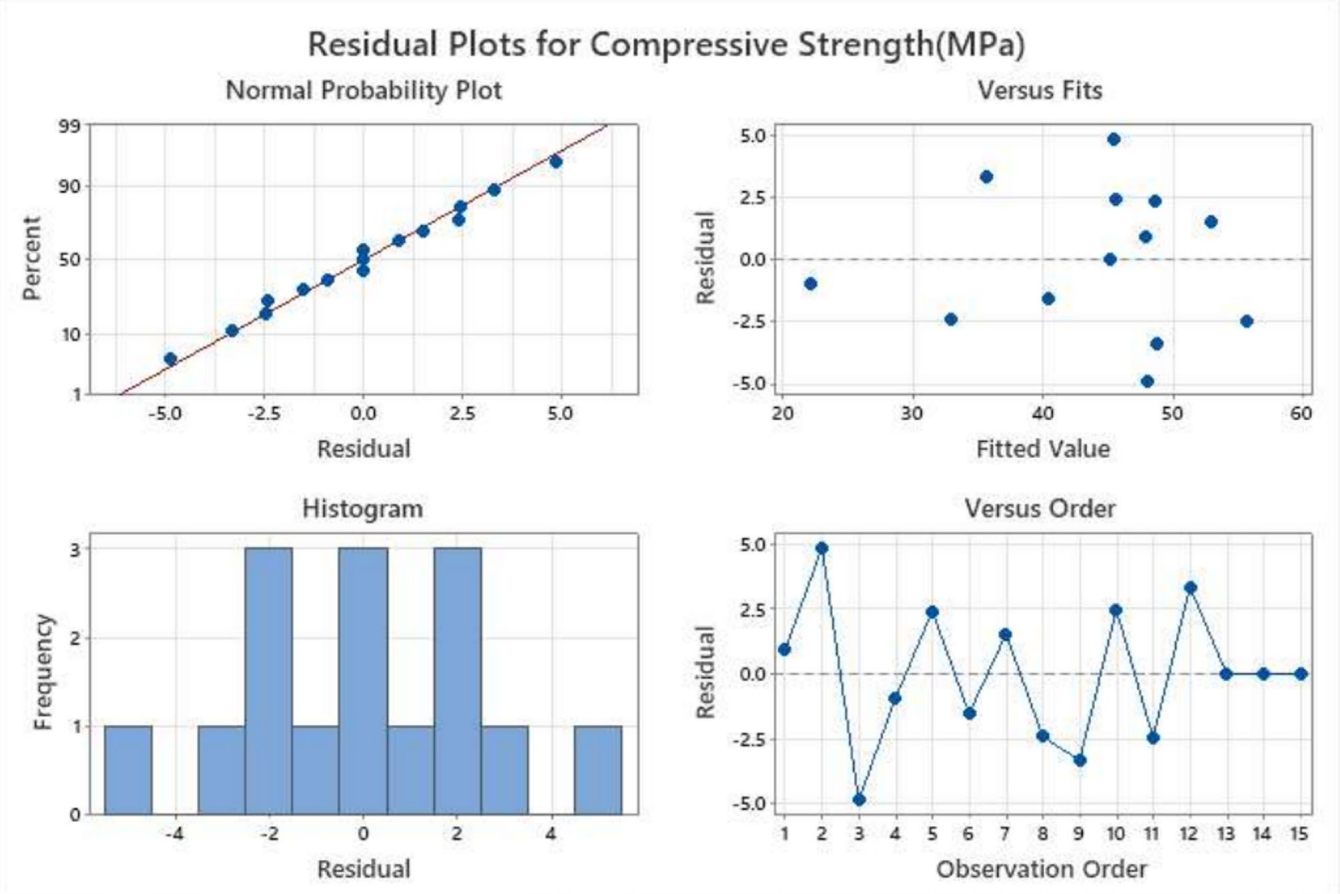
Residual diagnostic plots for compressive strength response showing (**a**) normal probability plot, (**b**) residuals versus fitted values, (**c**) histogram of residuals, and (**d**) residuals versus observation order.

Residual analysis was carried out to verify the statistical validity of the developed model for flow, IST, FST, and Compressive strength. The residual plots consist of normal probability plots, residuals versus fitted values, histograms, and residuals versus observation order, as shown in Figs. 6, 7, 8 and 9.

##### Normal probability plot

The normal probability plots for all four responses show that the residuals are closely aligned along the straight reference line, indicating that the error terms are normally distributed for the all parameters. This alignment confirms the reliability of the predictions underlying the RSM, particularly the normality of residuals, which is critical for accurate model interpretation. This confirms that the regression model’s normality assumption is satisfied. Only minor deviations at the tails are observed, which are common in experimental datasets and do not significantly affect the model reliability, which can be seen in Figs. 6, 7, 8 and 9.

##### Residuals versus fitted values

The plots show a random scatter of points around the zero reference line without any systematic pattern or funnel-shaped distribution. This indicates that the residual variance remains approximately constant across predicted values, confirming stable variance across mixes.

##### Histogram of residuals

The histograms of the residuals exhibit approximately symmetric bell-shaped distributions centred near zero. This further supports the normal distribution of errors and indicates that the prediction errors are randomly distributed.

##### Residuals versus observation order

The plots of residuals against observation order display no discernible trends or systematic sequences, indicating that the residuals are independent of the experimental run order.

The lack of curvature or departure from the line indicates that the variance of errors (residuals) is constant for all levels of an independent variable. Furthermore, there are no points that are considered to be outliers, which is an indication of the validity of the experimental results and the use of the Box-Behnken Design (BBD) optimised mixes. Despite a wider residual spread, the linearity confirms the statistical validity of the RSM model for flow behaviour. Though residuals vary widely (± 50), no significant curvature is observed, indicating a good model fit. Minor deviations seen at the extremes suggest slight variation, likely due to sensitivity to activator content.

#### Model adequacy and statistical analysis

**Table 4:** Single summarised ANOVA for all responses.

Response	Model DF	F-value	*p*-value (Model)	*R*² (%)	Adj-*R*² (%)	Pred-*R*² (%)	Std. Dev (S)	Lack-of-Fit (*p*)
Compressive strength (MPa)	9	5.56	0.037	90.91	74.55	0.00	4.44	> 0.05
Flow (mm)	9	1.09	0.490	66.19	5.33	0.00	6.42	> 0.05
Initial setting time (min)	9	1.52	0.335	73.27	25.14	0.00	20.79	> 0.05
Final setting time (min)	9	1.72	0.285	75.62	31.74	0.00	33.43	> 0.05

The appropriateness of the developed models was evaluated using Analysis of Variance (ANOVA), coefficients of determination (R²), adjusted R², lack-of-fit tests, and residual diagnostics.

Table 4 presents the ANOVA results for Compressive strength, flow, initial, and final setting time, developed using a quadratic RSM model.

For compressive strength, the model was statistically significant (*p* = 0.037) with a coefficient of determination (R² = 90.91%), indicating that the independent variables had a combined influence on strength development. The lack-of-fit was statistically insignificant (*p* > 0.05), confirming that the selected quadratic model appropriately represents the experimental data within the investigated domain.

In contrast, for flow, Initial and final setting time, the models were statistically non-significant (*p* > 0.05). Though the R^2^ values appear moderately high(66–76%), the adjusted R^2^ values were considerably lower, indicating that the inclusion of multiple model terms relative to the number of experimental runs reduced the effective explanatory power. This suggests that the independent variables did not exert a strong measurable influence on these responses within the selected experimental range.

The lack-of-fit tests for all responses were found to be statistically insignificant (*p* > 0.05), thus confirming that the developed quadratic models adequately represent the experimental data. The BBD design incorporated three centre-point replicates to estimate pure experimental variability. The replicate data were utilised in the ANOVA framework in Minitab to calculate lack-of-fit and assess the reliability of the developed quadratic models. Furthermore, residual plots and normal probability plots also show random scatter and approximate normality, thus validating the assumptions of regression analysis.

Notably, the Pred R^2^ values were zero for all responses. Predicted R² is calculated based on the PRESS (Predicted Residual Error Sum of Squares) statistic and reflects the model’s ability to predict new experimental observations. In the present study, this behaviour can be attributed to the relatively small variation in responses (particularly consistency and flow) and the sensitivity of the PRESS statistic to minor prediction errors. Consequently, although the developed models exhibit acceptable goodness-of-fit(R^2^) and a non-significant lack of fit, their predictive capability for new observations is limited. Therefore, the developed models are considered valid within the investigated design space and should be interpreted only within the range of input variables considered for this study (i.e., interpolation within the experimental domain), where they adequately represent the relationships between variables and responses. However, their predictive capability outside this range is limited (i.e., extrapolation beyond the investigated range is uncertain), and the models should not be considered for prediction beyond the studied design space.

##### Optimisation and desirability function analysis

The desirability function method was employed for numerical optimization to find the optimal combination of binder content, alkali activator dosage, and silica fume content that simultaneously satisfies multiple performance criteria. The goal of the optimization was to increase compressive strength, while maintaining sufficient flow and practical setting times suitable for on-site applications.

The total desirability was calculated as the geometric mean of the individual desirability’s after each response was converted into an individual desirability function ranging from 0 (undesirable) to 1 (fully desirable). Equal importance was assigned to all responses to prevent the results from being biased towards any one performance parameter. The optimization search was limited within the ranges of the factors that were experimentally investigated to ensure practical relevance and model reliability.

The desirability-based optimization process yielded a wide optimal region instead of single unique solution, suggesting robustness of the developed models. The optimal region was identified as a region of moderate binder content, controlled alkali activator dosage, and intermediate to higher silica fume content, where high compressive strength was achieved without compromising flowability or causing excessively rapid or delayed setting. This behaviour is consistent with the interaction effects observed in the response surface plots, where silica fume contributed to balanced reactivity and improved workability control.

The existence of overlapping optimal regions across multiple responses shows the suitability of the RSM approach for multi-objective optimisation of one-part alkali-activated binders. However, because of experimental variability, slight differences between expected and experimental values are anticipated and the limited number of design points inherent to Box–Behnken designs, the optimised solutions fall well within acceptable engineering limits, confirming the practical applicability of the proposed mix design framework.

The optimised solutions were further validated by comparing predicted and experimental responses, which established good agreement with low prediction errors, confirming the adequacy of the optimisation procedure.

### Optimisation and validation

In this study, optimisation was primarily based on the 28-day compressive strength. The preliminary multi-response optimisation, which included fresh properties as constraints, significantly lowered the predicted compressive strength, limiting it to around 43 MPa. However, several experimental mixtures within the design matrix achieved compressive strengths of up to 54 MPa. Therefore, the optimisation was conducted with the aim of maximising compressive strength. The fresh properties of the optimised mixture were then experimentally verified to ensure acceptable workability and setting behaviour. Consequently, the following ratio was determined to be optimal, and the predicted value is quite dependable.

Table 5 summarises the complete RSM experimental design matrix, along with all measured fresh and mechanical responses for each run.

**Table 5:** Experimental matrix and measured responses of RSM runs.

Runs	FA (%)	GGBS (%)	SF (%)	Alkali activator (%)	Consistency (%)	Flow (mm)	IST (mins)	FST (mins)	28-D CS (MPa)
1	52.5	40	7.5	10	27	180	79	388	48.88
2	72.5	20	7.5	10	27	170	70	372	50.38
3	52.5	40	7.5	14	25	185	72	298	43.16
4	72.5	20	7.5	14	26	170	88	300	21.26
5	55	40	5	12	26	175	75	370	50.99
6	75	20	5	12	27	170	126	385	38.86
7	50	40	10	12	25	180	60	380	54.52
8	70	20	10	12	26	180	110	410	30.57
9	65	30	5	10	25	190	133	365	45.49
10	65	30	5	14	25	180	85	420	48.04
11	60	30	10	10	26	170	80	375	53.31
12	60	30	10	14	25	180	60	305	38.94
13*	62.5	30	7.5	12	25	185	60	322	45.15
14*	62.5	30	7.5	12	25	185	60	322	45.15
15*	62.5	30	7.5	12	25	185	60	322	45.15

*Centre point replicates.

Table 6 shows the results of compressive strength, actual and predicted values as per RSM, according to the optimisation’s goal. The prediction error is less than 10% across the 14 runs compared with the actual value used to optimise the OPAAB mix proportions, indicating a good level of model accuracy. Response prediction values for compressive strength were derived using individual response models, and the model was validated by calculating the absolute deviations from the actual values.

**Table 6:** Model verified results showing predicted and experimental 28-day compressive strength of OPAAB with percentage error.

Responses	Mix No	Binder FA (%)	GGBS (%)	Alkali activator (%)	SF (%)	Predicted values (MPa)	Experimentally obtained (MPa)	% of error
Compressive Strength (MPa)	M1	52.5	40	10	7.5	47.96	48.88	1.8
	M2	72.5	20	10	7.5	45.54	50.38	9.6
	M3	52.5	40	14	7.5	47.99	43.16	11.1
	M4	72.5	20	14	7.5	22.17	21.26	4.2
	M5	55	40	12	5	48.59	50.99	4.7
	M6	75	20	12	5	40.38	38.86	3.9
	M7	50	40	12	10	52.99	54.52	2.8
	M8	70	20	12	10	32.96	30.57	7.8
	M9	65	30	10	5	48.80	45.59	7.0
	M10	65	30	14	5	45.59	48.04	5.0
	M11	60	30	10	10	55.75	53.31	4.5
	M12	60	30	14	10	35.62	38.94	8.5
	M13	62.5	30	12	7.5	45.15	45.15	–
	M14	62.5	30	12	7.5	45.15	45.15	–
	M15	62.5	30	12	7.5	45.15	45.15	–

The higher error observed in Run 3 corresponds to a slight over-prediction by the model, though the absolute deviation remains within acceptable limits for RSM-based optimisation. Absolute percentage error values are reported as positive magnitudes, irrespective of over- or under-prediction. Although a zero error is observed at the design centre, the overall mean absolute percentage error (MAPE) remains within acceptable limits, confirming good predictive performance of the model.

#### Mean Absolute Percentage Error (MAPE)

Using the absolute percentage errors above:

$$\:\mathrm{MAPE}=\frac{1}{n}\sum\:\mid\:\frac{{Y}_{\mathrm{exp}}-{Y}_{\mathrm{pred}}}{{Y}_{\mathrm{exp}}}\mid\:\times\:100$$

Where: n=total number of experiment runs in BBD, Y_exp_= experiment value/actual value, Y_pred_= predicted value from the model.

#### Calculated MAPE ≈ 5.4%

The developed RSM model showed good predictive capability, with a mean absolute percentage error (MAPE) of approximately **5.4%**, indicating close agreement between predicted and experimental compressive strength values.

Table 7 shows the optimal parameter values obtained using the BBD-RSM method. The model validation was carried out with the optimal mixture predicted by the response optimiser within the Response Surface Methodology framework. Since the optimisation identified a single optimal combination of independent variables aimed at achieving maximum compressive strength, validation was performed at this single predicted condition. The experimental compressive strength for the validation mix closely matched the predicted value, demonstrating the model’s satisfactory predictive ability within the studied design domain. Fresh properties were also evaluated to confirm acceptable workability and setting behaviour of the optimised mixture.

The optimised mix yielded a composition of 56.00% fly ash, 34.00% GGBS, 10% alkali activator, and 10% silica fume. At this combination, the model predicted a maximum compressive strength of 57.00 MPa, achieving a composite desirability value of 1.000.

**Table 7:** Optimum mix based on the BBD-RSM method.

Responses	Predicted	Experimental	% Error
Consistency (%)	–	26	–
Flowability (mm)	–	168	–
Initial Setting time(min)	–	78	–
Final Setting time (min)	–	370	–
CS 28 days (MPa)	57.00	53	7.0

To validate the model prediction the optimum mix was prepared and tested. The optimised mix experimentally exhibited compressive strength of 53 MPa, resulting in a prediction error of 7.0%. The experimental value falls within the 95% prediction interval (41.20,72.80 MPa), confirming satisfactory agreement between the predicted and experimental values.

Although fresh properties were not as optimisation constraints, flow, IST, FST were evaluated experimentally to verify practical applicability. The obtained values (Flow = 168 mm, IST = 78 min, FST = 370 min) were within acceptable operational limits, demonstrating that the optimized mix satisfies both mechanical and workability requirements. The close agreement between predicted and experimental strength confirms the adequacy of the developed RSM model for strength optimization.

The successful validation demonstrates that compressive strength can be reliably optimized using RSM, even when fresh property models exhibit comparatively lower predictive capability.

#### 3D surface plots

**Fig. 10:**
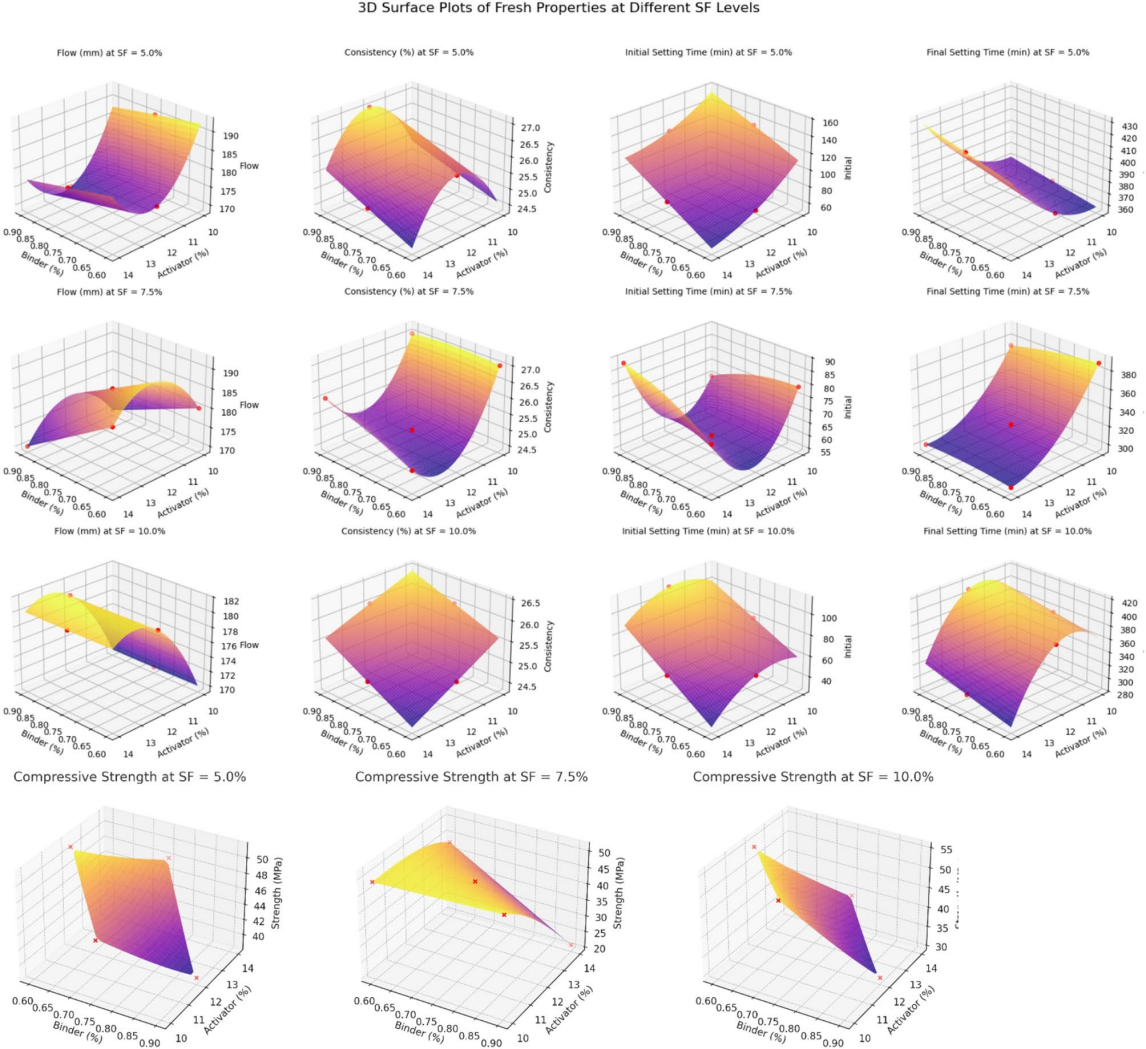
3D surface plots of OPAAB.

3D surface plots obtained for optimised mixes through BBD-RSM provide valuable insights into the combined effects of how two independent variables (e.g., binder content and activator dosage) interact to influence a response variable (e.g., flowability, consistency, or setting time) while keeping the third factor (SF level) constant, as shown in Fig. 10. The X- and Y-axes represent two independent factors (activator and binder %). The Z-axis reflects the predicted response (e.g., flow). The surface shows how the response changes when varying both factors together, for varying silica fume percentage, revealing interaction effects and the presence of local maxima or minima (optima or sub-optima) for performance targets^60^.

These plots assist in identifying optimal mix proportions, understanding factor interactions, and guiding further experimental design by highlighting regions of sensitivity and trade-offs between responses.

##### Flow behaviour

From Fig. 10, we observe that at 5% SF levels, the flow showed a slight decreasing trend with increasing binder (fly ash %) content and activator dosage, indicating reduced workability due to higher reactivity and particle packing. At 7.5% SF, a non-linear response was noticed, where moderate binder and activator contents resulted in maximum flow, signifying an optimal point between solid loading and efficiency of activation. At 10% SF, flow values were relatively stable but slightly lower compared to 5 and 7.5% SF contents, implying that excessive SF can hinder workability due to increased water demand and surface area, stressing the need for balance between reactive and inert fillers in the mix.

##### Consistency

From Fig. 10, we can see the consistency values varied between 25% and 27% for all levels of silica fume (SF) levels. At low SF content (5%), consistency showed a slight declining trend when binder or activator dosage increased. Conversely, as SF increased to 7.5% and 10%, the system became more stable, and the consistency values became more uniform. This might be due to the filler effect of silica fume and its ability to densify the paste matrix, minimising the variation in water demand. The slight drop in consistency at higher binder levels could be related to improved packing and reduced pore space, which decreases binder fluidity.

##### Setting time

The interaction plots of RSM show a significant coupled effect between silica fume (SF) dosage and activator dosage on both initial and final setting times. At lower SF levels (5%), an increase in activator level leads to substantial acceleration in setting time due to high availability of alkali and fast geopolymerisation kinetics. In contrast, at higher SF content (10%), the interaction surfaces flatten and setting times become less dependent to activator dosage. The interaction behaviour of these variables indicate that silica fume moderates alkali reactivity by rapidly consuming alkali ions and providing abundant nucleation sites, thereby reducing localised alkalinity and preventing premature gel precipitation. Such moderation of reaction kinetics results in a more stable and controllable setting window, as commonly observed in silica-rich alkali-activated binders.

##### Compressive strength

As shown in Fig. 10, at the 5% SF level, the compressive strength showed a relatively uniform distribution across the studied range of binder and activator contents, with values ranging from nearly 45 to 59 MPa. The binder content increase led to a marginal reduction in strength while the system remained stable. This suggests that at lower SF levels, the binder and activator combination produces a consistent paste matrix with minimal interaction effects, likely due to limited microstructural refinement. At 7.5% SF, a moderate silica fume content led to more pronounced variations. Compressive strength decreased significantly with increasing activator dosage in mix 4, even at high binder contents. This may be attributed to excessive alkalinity, which can accelerate reaction kinetics. Despite this, the middle zone of the plot (moderate binder and activator) still maintained compressive strength above 45 MPa, indicating a partial balance in geopolymer gel formation. The compressive strength at SF = 10%, an optimal strength zone was clearly visible at intermediate binder and activator levels, with peak compressive strength reaching above 54 MPa. This trend shows that 10% SF greatly aids in the development of strength through Pozzolanic reaction, Filler effect, and Improved particle packing. However, excessive activator content can still deteriorate mechanical performance, underscoring the importance of balanced mix proportions.

### Microstructure analysis

#### SEM and EDS analysis

**Fig. 11:**
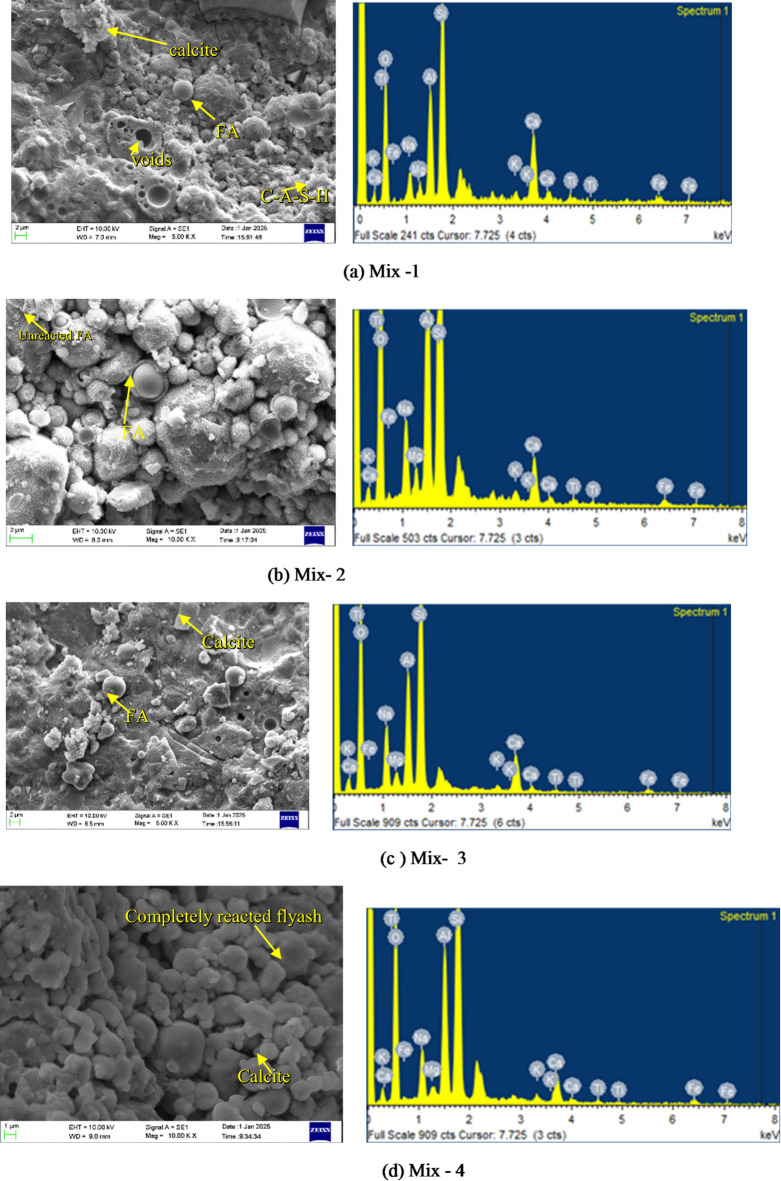

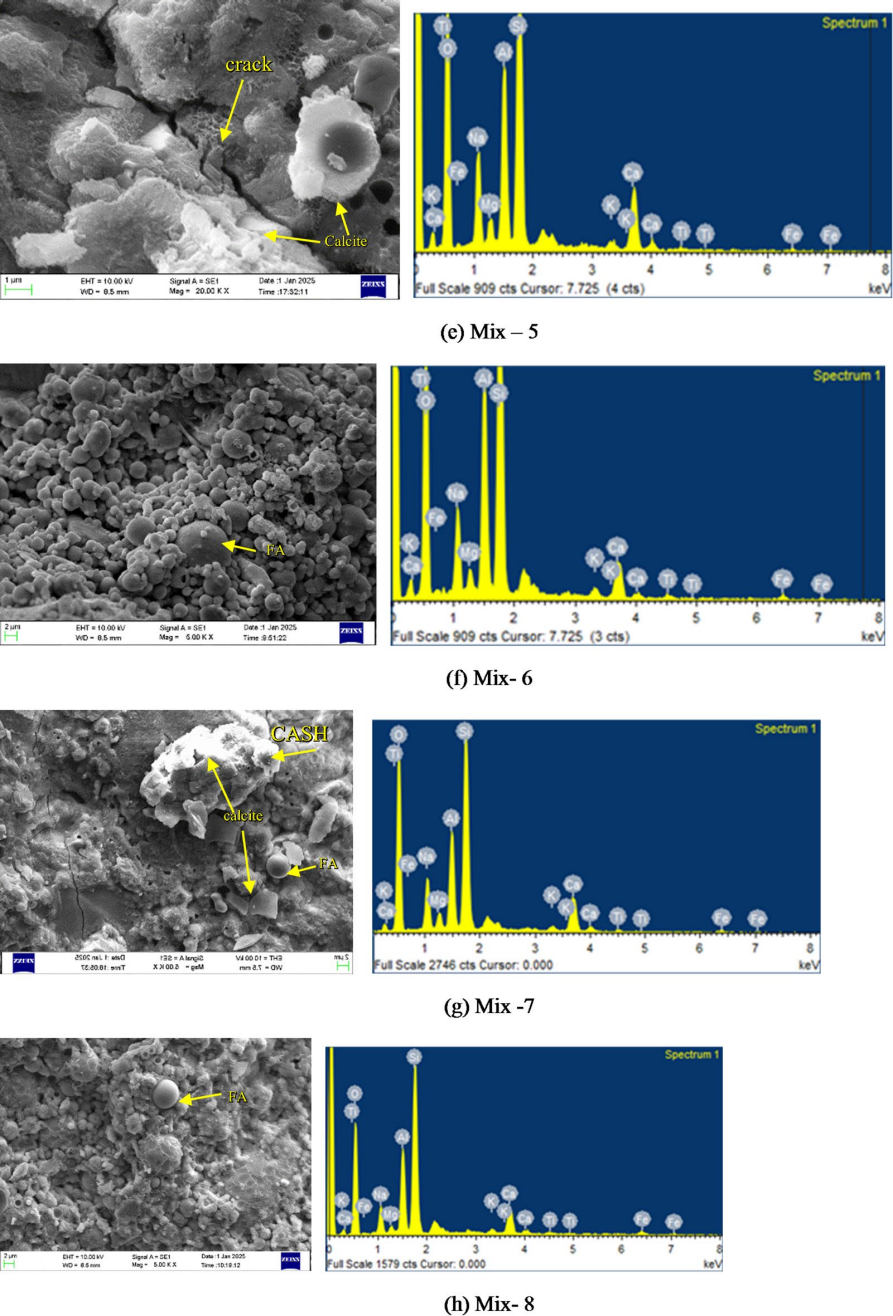

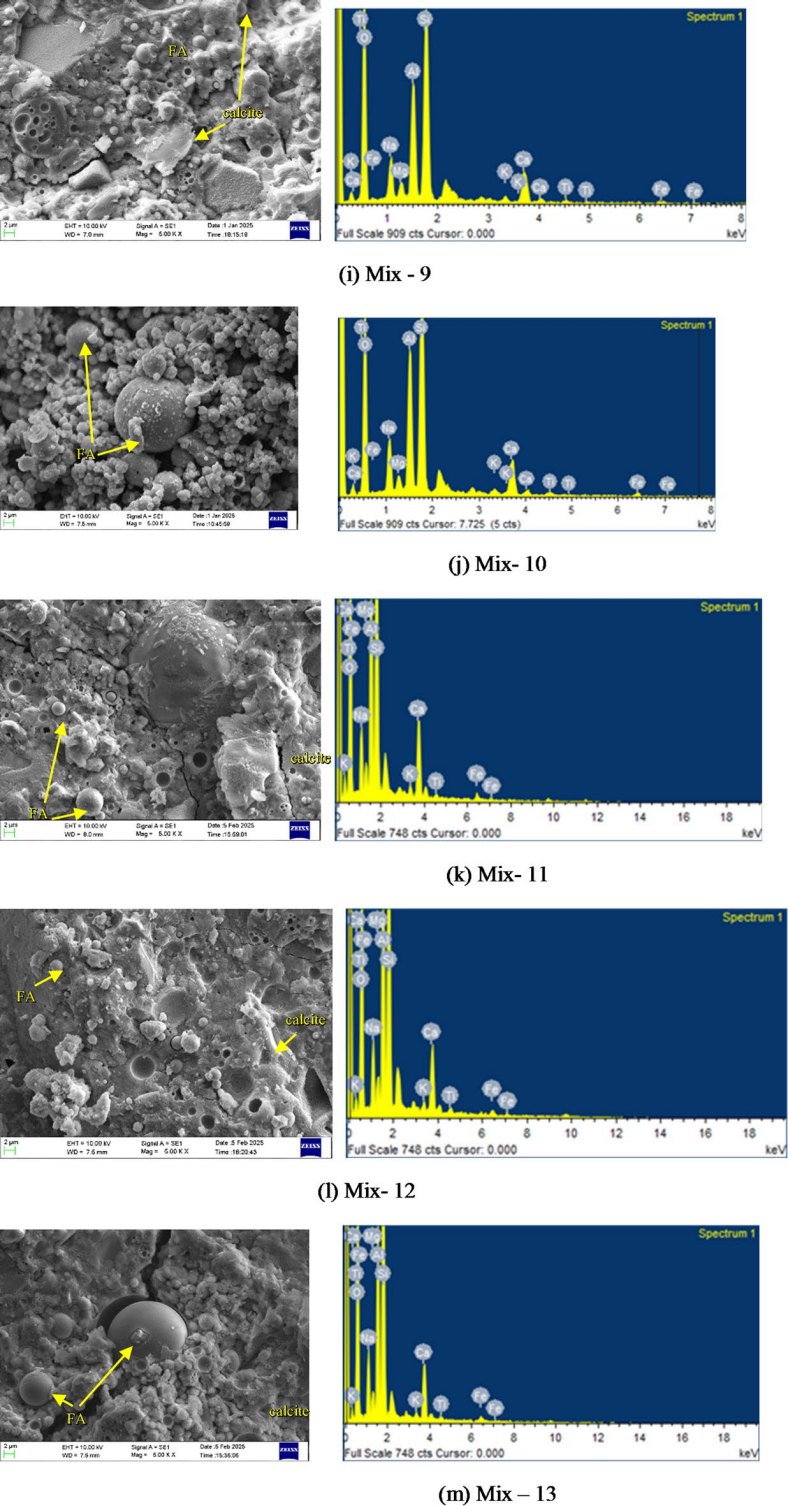
SEM images of OPAAB mixes.

To understand the reaction mechanisms and microstructural evolution in one-part alkali-activated binder, a combined SEM and EDS investigation was conducted on all mixes after 28 days of curing in ambient air. The SEM micrographs revealed varying degrees of geopolymerization, gel development, and morphological characteristics, while EDS spectra confirmed the elemental compositions associated with alkali activation and gel formation are presented in Fig. 11.

Silica fume plays a key role in driving the reactions and shaping the microstructure of one-part alkali-activated pastes. In Mix M1 (Fig. 11(a)), we observed a dense microstructure featuring C-A-S-H gel, a few unreacted fly ash, voids and partially reacted GGBS. EDS reflects the highlighted strong Si, Al, Ca, and O signals (with moderate Na), pointing to a hybrid mix of N-A-S-H and C-A-S-H gels supported by high calcium for solid C-A-S-H and an ideal Si/Al ratio for better cohesion. Mix M2 (Fig. 11(b)) featured unreacted fly ash and voids, signalling limited geopolymerization and a loose matrix with weak structure. This delayed substantial C-A-S-H gel formation early on, but strength developed in later stages. EDS showed prominent Si, Al, Fe, and Na, but reduced Ca intensity. Mix M3 (Fig. 11(c)) displayed a favourable microstructure, with less unreacted fly ash resulting in a tightly packed matrix of C-A-S-H gel, and GGBS particles well integrated. Its EDS showed Si, Al, Ca, and O peaks, evidence of strong reaction products. Mix M4 (Fig. 11(d)) was highly porous, with abundant unreacted fly ash and formation of gel precipitation. Mix M6 (Fig. 11(f)) had large amounts of unreacted fly ash, leading to incomplete geopolymerization; EDS confirmed low Ca and higher Fe/Ti in residual phases, limiting cohesion. Whereas in Mix 7 (Fig. 11(g)), we can see abundant C-A-S-H gel in the form of foil-like, amorphous formations, likely blended with N-A-S-H from fly ash, forming a cohesive matrix that effectively bridges particles. Most spherical fly ash grains have reacted edges, while angular GGBS fragments blend seamlessly. Minor voids and thin cracks hint at slight shrinkage or air entrapment, but overall porosity stays low. The spectrum overlays confirm dominant Si, Al, Ca, and O signals (high Ca/Si ratio ~ 1.5–1.9), with moderate Na and traces of Fe/Ti validating robust hybrid gel development without excess unreacted phases. This dense, gel-rich structure explains the exceptional strength, surpassing typical blends by reducing defects and enhancing cohesion. Mix M8 (Fig. 11(h)) showed a heterogeneous structure, partial fly ash reaction, voids, and C-A-S-H deposits with consistent EDS elements but lower Ca, suggesting incomplete reactions and reduced matrix cohesion. Mixes M9 (Fig. 11(i)) echoed this pattern, delivering dense matrices with partial fly ash reaction and clear C-A-S-H presence. Mix M10 (Fig. 11(j)) displayed unreacted fly ash particles, with texture showing air entrapment or poor mixing, and EDS revealed Si, Al, Ca (minor Na, Fe), but poor product integration. Mixes M5 (Fig. 11(e)) and M11-M15 (Fig. 11(k, l,m)) offered compact matrices with evident C-A-S-H gel and minor cracks likely from autogenous shrinkage or curing stresses, but backed by strong Si, Al, and Ca in EDS, confirming reaction product.

#### XRD analysis

**Fig. 12:**
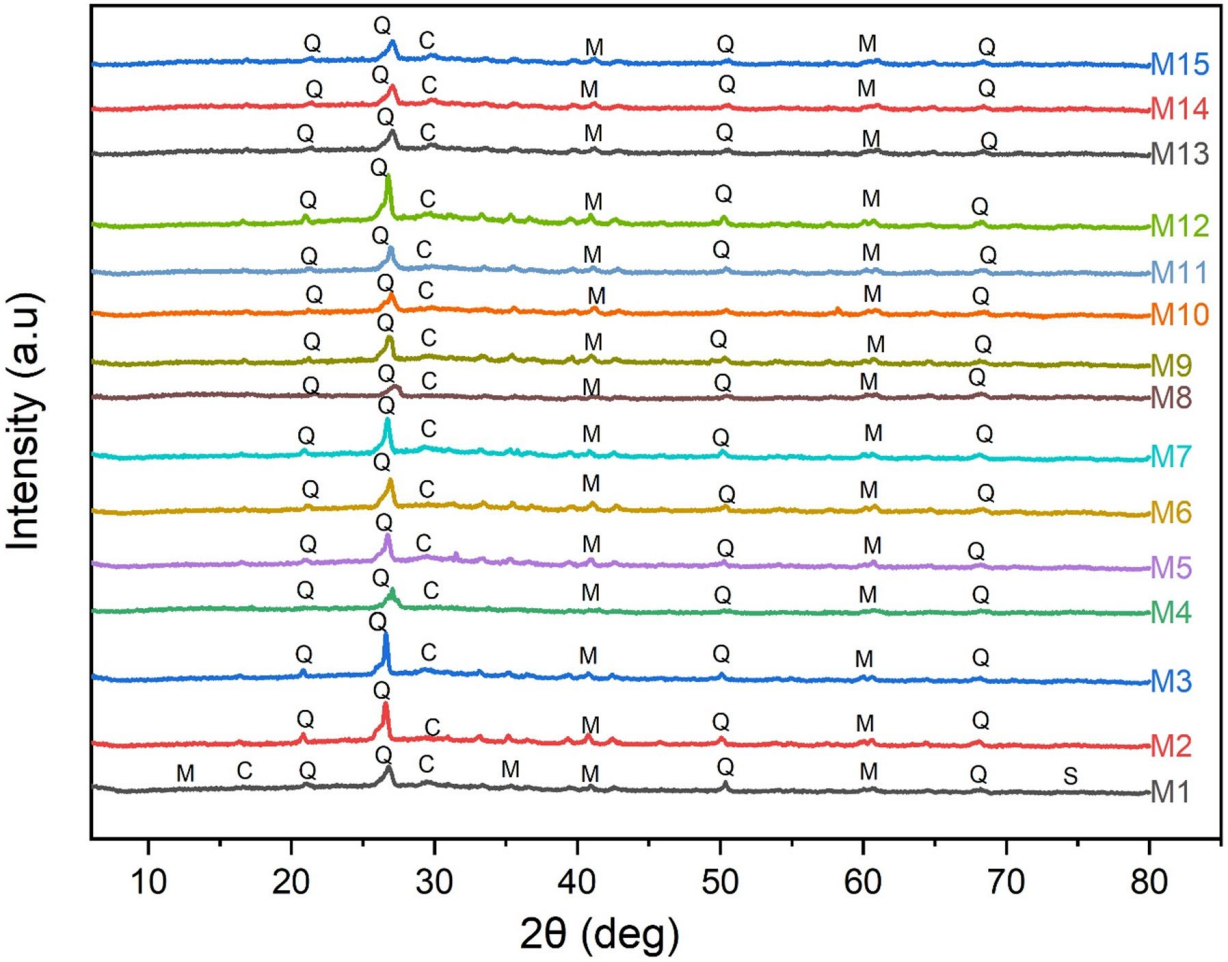
XRD Images of OPAAB mixes.

Figure 12 displays the XRD patterns of the OPAAB mixes. The XRD patterns of all 15 mixes (M1–M15) exhibit a combination of crystalline and amorphous phases.

The major crystalline phases identified across most samples include Quartz (Q), Calcite (C), and Mullite (M). Quartz peaks are dominant near 2θ = 26.6°, confirming the presence of unreacted silica from the fly ash and GGBS. The persistent presence of mullite, a stable phase originating from calcined aluminosilicates in fly ash, shows incomplete reaction of some precursor materials^61^. Calcite formation is likely due to carbonation of free lime and reaction products, especially in mixes with higher GGBS content^62^.

Notably, mix M1 shows additional peaks attributable to residual sodium silicate (S), indicating incomplete dissolution of the solid activator in the early phase stage. This phase gradually diminishes in later samples, suggesting enhanced activator reaction and incorporation into reaction products. To enable a more systematic comparison, the OPAAB mixes were grouped based on similarities in diffraction features. Mixes M1–M2 show strong crystalline reflections of quartz and mullite, along with residual sodium silicate peaks in M1, indicating incomplete activator dissolution and limited early-stage geopolymerization.

Mixes M3–M9 show a gradual reduction in crystalline peak intensity accompanied by a broader amorphous hump in the 20°–40° 2θ region, suggesting improved dissolution of fly ash and GGBS and progressive development of aluminosilicate gel. In contrast, mixes M10–M15 exhibit the most prominent amorphous halo with comparatively reduced crystalline peaks, indicating enhanced polycondensation and formation of a well-developed geopolymeric binder matrix.

This evolution in phase correlates qualitatively with the increase in compressive strength observed in several mixes. The mechanical performance of alkali-activated binders is governed by the formation and continuity of amorphous reaction gel (Table 8).

**Table 8:** Grouped XRD features and qualitative strength correlation of OPAAB mixes.

Mix group	Key XRD characteristics	Reaction implication	Strength trend
M1–M2	Strong quartz/mullite peaks; residual sodium silicate	Limited dissolution and gel formation	Low
M3–M9	Reduced crystalline peaks; moderate amorphous hump	Progressive geopolymerization	Moderate/High
M10–M15	Dominant amorphous halo; minimal crystalline residues	Enhanced gel network formation	Moderate

#### FTIR analysis

**Fig. 13:**
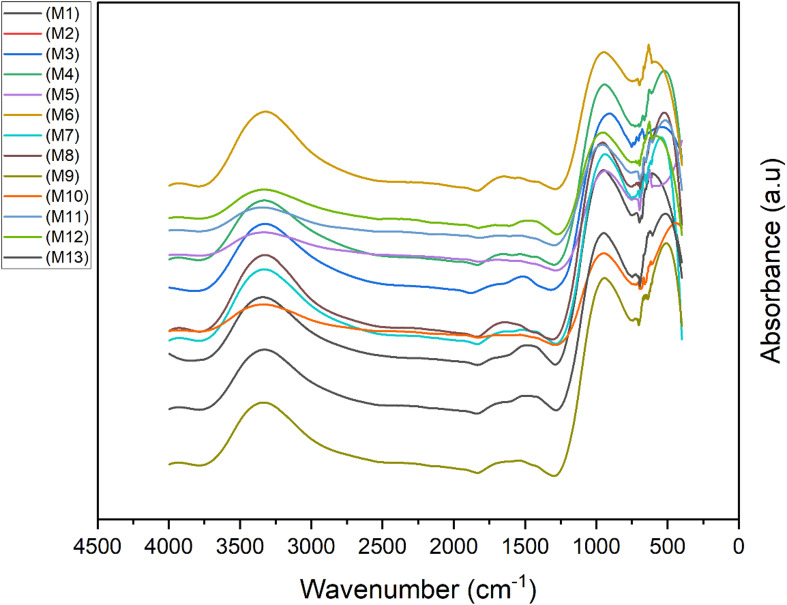
FTIR of OPAAB mixes.

As displayed in Fig. 13, the Fourier Transform Infrared (FTIR) spectra of the OPAAB provide clear evidence of aluminosilicate gel formation and the role of silica fume in governing the reaction mechanism. All mixes exhibit broad bands in the region of 3200–3600 cm⁻¹, corresponding to O–H stretching vibrations of bound water associated with hydration and geopolymer gel formation, while the band near 1640 cm⁻¹ is attributed to H–O–H bending vibrations, indicating physically and chemically bound water within the reaction products.

In the FTIR spectra, the most prominent changes occur in the so-called fingerprint region (400–1200 cm⁻¹), which indicates the vibrations of the aluminosilicate framework. Before activation, the raw precursors exhibit asymmetric stretching vibrations of Si–O–T bonds (T = Si or Al) at relatively high wavenumbers. After alkali activation, this band shifts noticeably downward to the 900–1000 cm⁻¹ range. This shift clearly indicates that Si⁴⁺ atoms in the tetrahedral network are being replaced by Al³⁺, which is a hallmark of geopolymerization. The lower frequency is due to Si–O–Al linkages having lower force constants than Si–O–Si bonds, confirming the formation of the characteristic sodium aluminosilicate hydrate (N–A–S–H) gel. New or intensified bands appearing around 463 cm⁻¹ and 910 cm⁻¹ further support the development of a well-connected aluminosilicate network, as these correspond to O–T–O bending vibrations in the formed gel structure. In pastes containing higher amounts of ground granulated blast-furnace slag (GGBS), additional shoulders and contributions from Ca–O–Si and Si–O–Al vibrations become visible. These indicate the simultaneous formation of calcium aluminosilicate hydrate (C–A–S–H) gel alongside the N–A–S–H phase. The presence of both gel types indicates a hybrid binding system, a common feature of alkali-activated materials when calcium-rich precursors such as GGBS are incorporated.

The incorporation of silica fume is evident from subtle shifts and increased intensities in the Si–O–T stretching bands, especially in the 900–1000 cm⁻¹ region. Under alkaline conditions, silica fume dissolves quickly, releasing highly reactive silicate species. These species take part in secondary polymerization reactions, enhancing cross-linking between the aluminosilicate chains and calcium-rich phases. As a result, the gel structure becomes more highly polymerized and homogeneous. This delayed yet stronger gel formation aligns with the typically lower early-age reactivity but superior long-term strength seen in silica fume-containing mixes.

These FTIR observations are in good agreement with SEM–EDS results, which show a dense, continuous gel matrix rich in Si, Al, Ca, and Na. The presence of these elements confirms the intergrowth of N–A–S–H and C–A–S–H gels, highlighting how silica fume promotes better matrix densification and microstructural uniformity.

In summary, the shifts and intensity changes in the FTIR spectra directly reflect a higher degree of geopolymerization, which underpins the improved mechanical performance of these optimized OPAAB.

#### TGA analysis

**Fig. 14:**
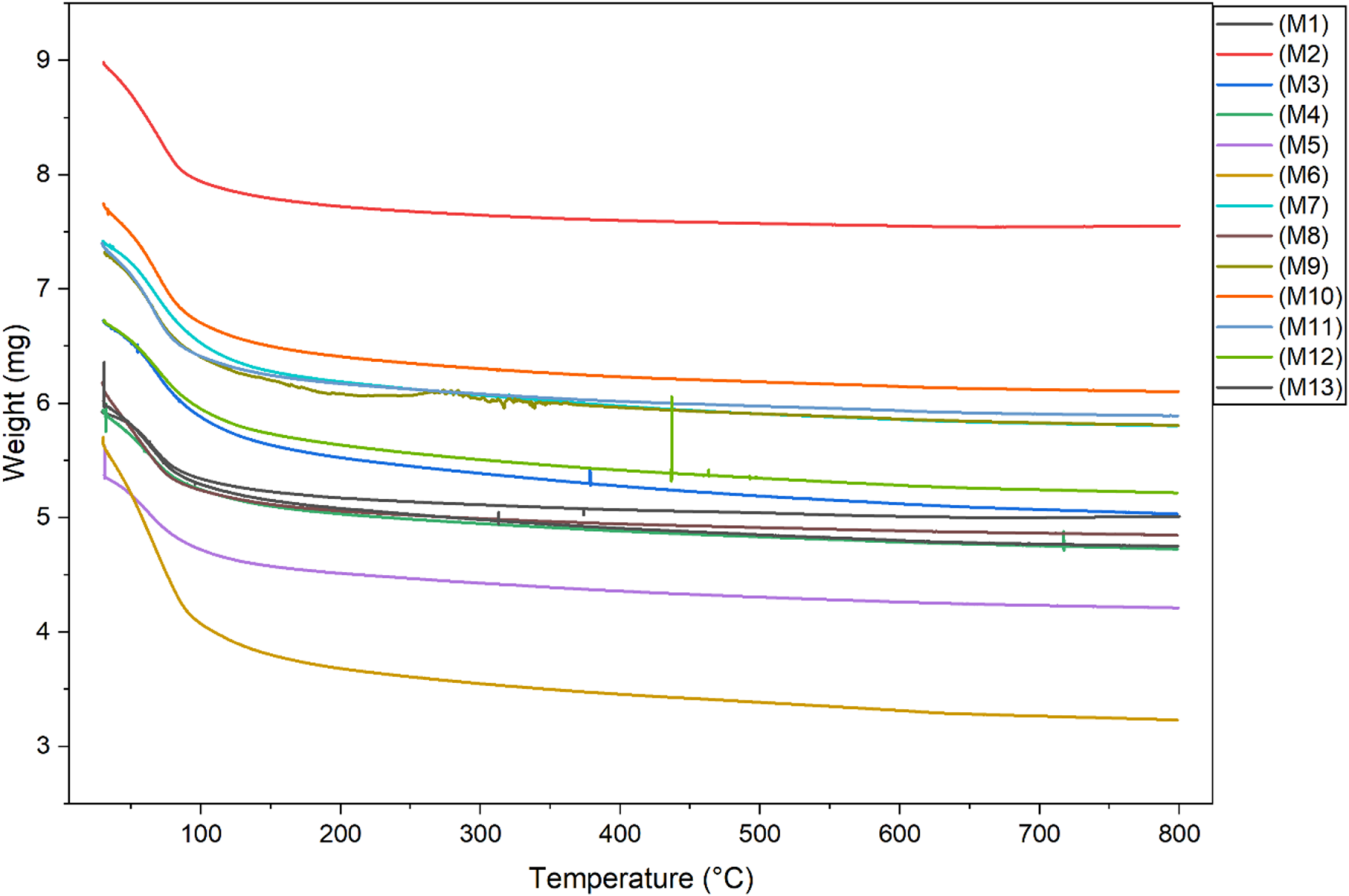
TGA analysis of OPAAB mixes.

The thermogravimetric analysis (TGA) results of alkali-activated binders at 28 days are presented in Fig. 14. All samples exhibit a mass loss between 20 °C and 100 °C, which corresponds to the evaporation of physically bound water. Notably, the total mass loss within this temperature range increases from approximately 3% to 5% for the binder activated with a higher alkali activator percentage.

The Non-Evaporable Water (NEW) serves as a semi-quantitative indicator of slag reactivity, as determined by Escalante-Garcia et al.^63,64^. Specifically, NEW was quantified using thermal analysis, as described by the following equation.

1 $${\mathrm{NEW}} = {\mathrm{1}}00*({\mathrm{weight}}_{{{\mathrm{12}}0^{ \circ } {\mathrm{C}}}} - {\mathrm{weight}}_{{{\mathrm{82}}0^{ \circ } {\mathrm{C}}}} )/{\mathrm{weight}}_{{{\mathrm{82}}0^{ \circ } {\mathrm{C}}}}$$

NEW = 100*(6.39–5.80)/5.80 = 10.17%.

The results show a NEW value of 10.17% for mix7, indicating that increasing the alkali content (Ac) enhances the reactivity of the Alkali-Activated Slag binder, as evidenced by the elevated NEW values. The thermogravimetric analysis (TGA) results further confirm these findings; the weight loss between 120 °C and 180 °C, linked to the dehydration of C-(A)-S-H, indicates increased hydration products with higher Activator^65^. Additionally, the weight loss between 300 °C and 400 °C, associated with hydrotalcite decomposition, also shows a positive correlation with increased activator concentration, reflecting heightened pozzolanic activity at 28 days after casting^66^. These observations align with previous studies, confirming that elevated activator levels promote early-age reactivity and binder formation.

### Environmental impact assessment

A life cycle assessment (LCA) was conducted to quantify the environmental impacts and carbon emissions of the mixes for 1 kg of cementitious materials, comparing them with those of the OPC-based binder system. A cradle-to-gate system was adopted. Previous similar studies have assessed the environmental impacts of OPC production using life cycle assessment approaches based on a functional unit of 1 kg of cement and cradle-to-gate system boundaries^67,68^(Table 9).

**Table 9:** EIA values for OPAAB mixes.

Material	Proportion (%)	CO₂ emission factor (kg/kg)	Total CO₂ (kg/kg binder)
Fly Ash	50	0.01	0.005
GGBS	40	0.05	0.020
Silica Fume	10	0.028	0.0028
Sodium Metasilicate	8	0.2	0.016
Sodium Hydroxide	4	1.5	0.060
Total	100	–	0.111
OPC Benchmark	100	0.9	0.9
Savings	–	–	87.7%

Assessment of environmental impact and cost implication (Table 10):

**Table 10:** CO_2_ emissions values.

Materials	Emission factors (kg Co_2_-eq/kg)
Flyash	0.011
GGBS	0.07
Silica fume	0.028
Sodium metasilicate	0.73
Sodium hydroxide	1.12
Water	0.000196
Cement	0.951

**Fig. 15:**
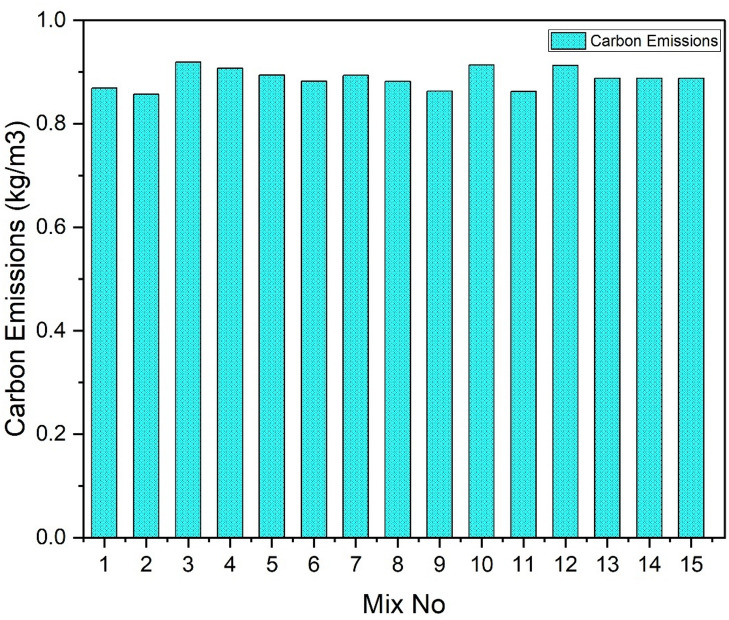
CO_2_ emission of total binder material per mix (per m^3^).

A cradle-to-gate life cycle assessment approach was adopted to quantify the total environmental impacts associated with the binder component of each mix (Fig. 15). The total cradle-to-gate ecological impact was calculated by summing the mass of each constituent multiplied by its corresponding life-cycle inventory emission factor for each impact category, including global warming potential (GWP), cumulative energy demand, water consumption, acidification, and eutrophication. Emission-factor sources are a combination of Ecoinvent-derived literature values and published LCA studies. The represented values in Table 10 are sourced from the literature Jagadisha et al.,2025^69^. Silica fume was modelled using literature-reported cradle-to-gate emission factors. Although silica fume is an industrial by-product, it was not assigned a strict zero-burden assumption; instead, the adopted emission factor reflects allocated upstream production impacts as reported in the selected LCA source. The alkali activator was assumed to be composed of sodium metasilicate and sodium hydroxide in a 2:1 mass ratio.

The OPAAB mix exhibited an emission factor of 0.111 kg CO₂-eq per kg of binder. For comparison, the environmental impact of OPC was assessed using the same functional unit and system boundaries, with a commonly reported emission factor of approximately 0.9 kg CO₂-eq per kg of cement^70^, which is consistent with values reported in cement life cycle assessment studies by the International Energy Agency and the Intergovernmental Panel on Climate Change. Based on this benchmark, the developed OPAAB binder demonstrates an 87.7% reduction in CO₂ emissions compared with OPC^71^.

The substantial reduction in emissions is mainly attributed to the utilisation of industrial by-products such as fly ash and ground granulated blast furnace slag (GGBS), which require significantly lower energy input compared to clinker production^72^. These findings align with previous life cycle assessment studies, indicating that alkali-activated binders exhibit considerably lower environmental impacts than traditional Portland cement systems.

Life cycle assessment (LCA) studies further reveal that AABs generally have embodied energy about 10–30% that of OPC^73,74^, despite the energy demand associated with alkali activators like sodium hydroxide and sodium silicate. However, even with activator energy considered, the net embodied energy remains substantially lower than OPC, emphasizing the environmental benefits. The environmental impact assessment of the alkali-activated slag binder shows its substantial sustainability advantages over OPC, supporting its suitability for eco-friendly construction applications^75,76^.

These findings align with recent literature demonstrating AAB as a green construction material with lower environmental impact and better long-term sustainability compared to traditional cementitious binders^77,78^. This assessment supports the advancement of AABs for broader adoption in sustainable building practices and merits inclusion in journal papers emphasizing sustainable material development^79^.

#### Eco-efficiency in relation to Compressive strength

**Fig. 16:**
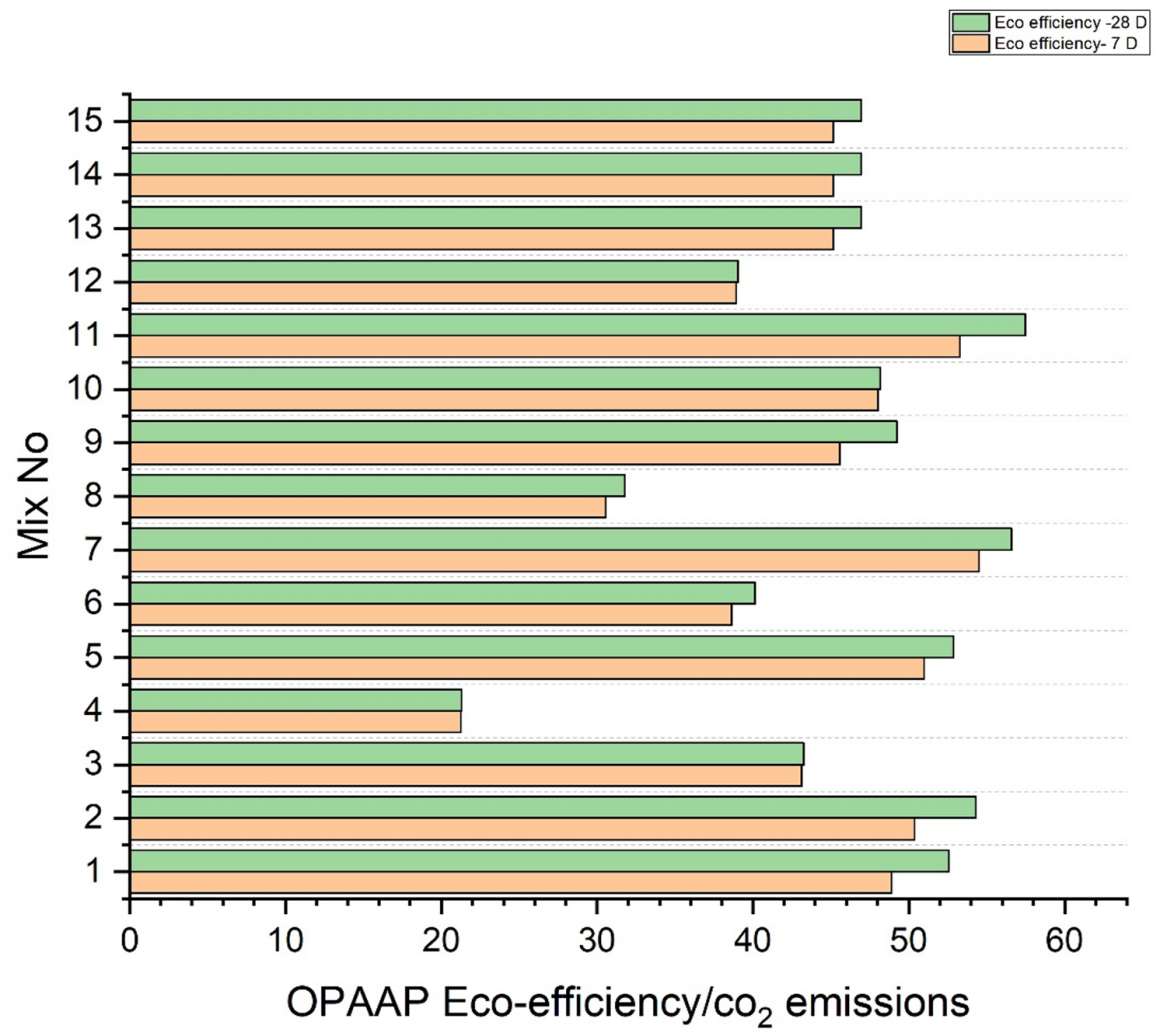
Eco-efficiency of Compressive strength/ CO_2_ emissions of OPAAB(MPa/g/CO_2_-eq/g).

Figure 16 shows the eco-efficiency of one-part alkali-activated binders evaluated at two curing ages, 7 days and 28 days, using the OPAAB metric normalised by CO₂ emissions. The eco-efficiency values for various mixes (Mix No. 1 to 15) show an overall increase from 7 to 28 days, indicating improved performance over time as the binder matures. For all mixes, eco-efficiency at 28 days exceeds that at 7 days, indicating a progressive improvement in binder efficiency with increasing curing age. Mixes 1, 7, 11, 13, 14, and 15 consistently demonstrate the highest eco-efficiency values at both curing intervals, with 28-day values exceeding 55, suggesting superior environmental performance coupled with mechanical or durability gains. Mix 4 shows the lowest eco-efficiency at both ages, around 20–25, indicating comparatively lower performance efficiency or higher emissions relative to others. The incremental increase in eco-efficiency from 7 to 28 days ranges roughly between 5 and 15 units across mixes, reflecting continuing hydration and strength development contributing to better environmental efficiency.

The observed trend of increased eco-efficiency with curing time underscores the significance of binder maturation in achieving optimal performance relative to carbon emissions. Higher eco-efficiency values denote that mixes are delivering greater functional output—likely in terms of mechanical strength or durability—for each unit of CO₂ emitted. The low eco-efficiency mix might suffer from suboptimal chemistry or excess activator use, which can increase emissions disproportionately, underlining the importance of mix design optimization. The general increase in efficiency upon extended curing also suggests potential for further gains through mineralogical transformations and microstructural densification over time.

In conclusion, this analysis verifies the environmental and technical feasibility of using one-part alkali-activated materials binders, with eco-efficiency improvements from 7 to 28 days indicating not only sustainability benefits but also the practical maturity of the material for construction applications.

## Conclusions

Based on the findings and discussions from the study on one-part alkali-activated pastes using the RSM-BBD- The binder is a ternary blend of low-calcium Class F fly ash, ground granulated blast furnace slag (GGBS), and silica fume, activated by solid sodium metasilicate and sodium hydroxide.

- One part ternary binder system consisting of blends of low-calcium Class F fly ash, ground granulated blast furnace slag (GGBS), and silica fume, combined with a solid alkali activator mixture of sodium metasilicate and sodium hydroxide lead to the formation of a stable and eco-friendly binder capable of achieving compressive strengths of approximately 53 MPa at 28 days under ambient curing conditions, demonstrating its suitability for structural applications.
- Response Surface Methodology (RSM) coupled with Box-Behnken Design enabled efficient, systematic optimisation of the binder composition and activator dosage. This method captured complex interactions among binder ratio, silica fume content, and activator dosage, allowing derivation of predictive models with less than 10% error and identifying an optimal blend that maximizes compressive strength (up to 54 MPa at 28 days) while maintaining good flow and practical setting times. This demonstrates the role of RSM in guiding formulation of one-part alkali-activated binders with tailor-made performance properties.
- This solid form binder retains comparable or superior mechanical properties with significant environmental benefits, including substantial CO₂ emission reductions (> 87%) by using industrial by-products and reducing processing energy.
- The statistically developed BBD-RSM model demonstrates the effectiveness of systematic mix design optimisation in developing a fly ash-dominant one-part alkali-activated binder with reliable mechanical performance (R² = 90.91%, *P* > 0.5).
- Silica fume contributes to pore structure refinement and improved particle packing, resulting in a denser microstructure that enhances strength.
- In conclusion, the carefully engineered binder properties, alongside single optimisation via BBD-RSM, culminate in a highly effective one-part alkali-activated binder system that is practical and sustainable for low-carbon construction applications. The synergy of silica fume and alkali activator dosages plays a pivotal role in optimising microstructure and mechanical properties, validating the superiority of the one-part system for modern, eco-friendly building materials.
- The present investigation successfully establishes statistically optimised mix proportions based on mechanical performance, but durability-related properties, which are critical for long-term structural applications, remain to be addressed.

## Data Availability

The data that support the findings of this study are available upon request from the authors.
